# Analysis of transcriptomic alterations induced by 33 different per- and polyfluoroalkyl substances (PFAS) in differentiated HepaRG cells

**DOI:** 10.1007/s00204-026-04429-5

**Published:** 2026-05-13

**Authors:** Heike Sprenger, Wiebke Alker, Anna Rocchi, Chiara Leo, Rosa Giglio, Greta Immobile Molaro, Francesco Dondero, Albert Braeuning, Thorsten Buhrke

**Affiliations:** 1 Department Chemical and Product Safety, German Federal Institute for Risk Assessment (BfR), Max-Dohrn-Str. 8-10, 10589 Berlin, Germany; 2 NGS and Bioinformatic Laboratory – Polo d’Innovazione di Genomica, Genetica e Biologia SRL, Strada del Petriccio e Belriguardo 35, 53100 Siena, Italy; 3 Department of Science and Technological Innovation, University of Eastern Piedmont Alessandria Novara Vercelli, Viale Michel 11, 15121 Alessandria, Italy

**Keywords:** PFAS, HepaRG, Transcriptomics, IPA, Nuclear receptors, Lipid metabolism, Cholesterol

## Abstract

Due to their high persistence and adverse health effects in humans the use of a number of per- and polyfluoroalkyl substances (PFAS) has been restricted. As a consequence, novel PFAS are increasingly being introduced for industrial applications, although toxicological data are still limited or lacking for many of these compounds. This study examined the molecular mechanisms of action of novel PFAS with a focus on mono- and polyether PFAS with linear or branched structures and either carboxylic acid or sulfonic acid functional groups. Differentiated HepaRG cells, a model of human hepatocytes, were exposed for 24 h to different PFAS congeners at three non-cytotoxic concentrations each. Total RNA was isolated and subjected to whole transcriptome analysis. The study provides transcriptomic data for in total 33 PFAS congeners, for 13 of them for the first time. For most PFAS, the number of differentially expressed genes (DEG) increased in a concentration-dependent manner, whereas five PFAS induced only minor transcriptional changes even at the highest test concentration. Ingenuity Pathway Analysis (IPA) revealed broadly comparable transcriptional responses across all 33 PFAS, indicating convergent molecular effects in HepaRG cells despite marked structural differences among the PFAS congeners. The tested PFAS consistently activated canonical pathways related to fatty acid and lipid metabolism, mainly regulated by the nuclear receptor PPARα, and also affected pathways related to xenobiotic metabolism, partially linked to PXR and CAR signaling. In addition, several PFAS inhibited cholesterol and bile acid biosynthesis pathways. IPA further predicted effects on hepatocyte-relevant upstream regulators such as HNF4A, HNF1A, and FOXA2. Finally, IPA tox-function analysis indicated associations between PFAS-induced transcriptional changes and liver diseases related to cholestasis.

**Supplementary Information:**

The online version contains supplementary material available at 10.1007/s00204-026-04429-5.

## Introduction

Per- and polyfluoroalkyl substances (PFAS) are a large group of chemicals used in the manufacture of numerous industrial and consumer products, including, e.g., water- and dirt-repelling coatings for cookware, papers and textiles (Gluge et al. 2020). Owing to their extraordinary chemical stability, many PFAS are resistant towards chemical or biological degradation and therefore persist in the environment (Cousins et al. 2020). Human exposure occurs mainly via contaminated water, food and air. Animal and epidemiological studies have shown that many PFAS are associated with adverse effects on the immune system, endocrine system, and liver (EFSA 2020; ATSDR 2021). However, the underlying molecular mechanisms of PFAS toxicity are still not fully understood. For liver-related effects, activation of peroxisome proliferator-activated receptor alpha (PPARα), a nuclear receptor involved in lipid metabolism, is a well-established molecular target of many PFAS (Behr et al. 2020b; Bjork and Wallace 2009; Wolf et al. 2008, 2012). In addition, legacy PFAS have been reported to affect xenobiotic metabolism pathways regulated by the pregnane X receptor (PXR) and the constitutive androstane receptor (CAR) (Rosen et al. 2017).

Because of their persistence and adverse health effects, the use of a number of PFAS including the two most prominent legacy congeners perfluorooctanoic acid (PFOA) and perfluorooctanesulfonic acid (PFOS) has been restricted under international and regional regulatory frameworks, including the Stockholm Convention on Persistent Organic Pollutants (POPs) (Stockholm Convention, https://chm.pops.int/Home/tabid/10001/Default.aspx) and the European Union POPs Regulation (EU 2019/1021; EU 2020/784), as well as under the broader REACH framework and related restriction measures (EU 2017/1000). As a result, industry has developed numerous alternative PFAS for industrial applications. Among others, many (poly)-ether PFAS have been introduced as replacements for compounds such as PFOA and PFOS. In these substances, the perfluorinated carbon backbone is interrupted by one or more ether oxygen atoms, distinguishing them structurally from the classic perfluoroalkylcarboxylic acid (PFCA) or perfluoroalkylsulfonic acid (PFSA) congeners. In contrast to well-characterized legacy PFAS, toxicological information is still limited or absent for many of these newer compounds. To compare PFAS-induced molecular effects in human hepatocytes, we performed a transcriptomic analysis in differentiated HepaRG cells exposed to 33 congeners, including 15 PFCA/PFSA congeners and 18 perfluoroalkylether carboxylic acid (PFECA) or perfluoroalkylether sulfonic acid (PFESA) congeners. The human hepatocarcinoma HepaRG cell line is a well-established in vitro model of human liver, and differentiated HepaRG cells constitute a mixture of hepatocyte- and cholangiocyte-like cells, in which the hepatocyte-like cells display morphological and biochemical features very close to primary human hepatocytes (Antherieu et al. 2010; Rogue et al. 2012). Transcriptomic profiling showed broadly similar response patters across all 33 PFAS, including consistent effect on lipid metabolism (PPARα-related) and xenobiotic metabolism (including PXR-related signaling). Additional changes related to cholesterol and bile acid metabolism, amino acid metabolism, and mitosis were observed only for a subset of the PFAS and predominantly at concentration near the cytotoxicity threshold.

## Materials and methods

### Chemicals

The 33 PFAS congeners were purchased at the highest available purity. 11-Chloroeicosafluoro-3-oxaundecane-1-sulfonic acid potassium salt (8:2 Cl-PFESA) was purchased from eNovation Chemicals (Green Brook, USA). 2,3,3,3-Tetrafluoro-2-[1,1,2,3,3,3-hexafluoro-2-(trifluoromethoxy)propoxy]-propanoic acid (Branched ADONA), methyl perfluoro-3,6,9-trioxatridecanoate (CH3-PFO3TriDA), perfluoroheptanesulfonic acid (PFHpS), perfluoro-3,6-dioxadecanoic acid (PFO2DA), perfluoro-3,6-dioxaheptanoic acid (PFO2HpA), perfluoro-3,6,9-trioxadecanoic acid (PFO3DA), perfluoro-3,6,9-trioxatridecanoic acid (PFO3TriDA ), ammonium 2-perfluoropentoxy-2,3,3,3-tetrafluoropropanoate (PFoxaOA), perfluoropentanesulfonic acid (PFPeS) and potassium perfluoro(4-methyl-3,6-dioxaoctane)sulfonate (similar to Nafion-BP2) were purchased from Apollo Scientific (Manchester, UK). 2,3,3,3-Tetrafluoro-2-(heptafluoropropoxy)propanoic acid (HFPO-DA), 2,3,3,3-tetrafluoro-2-(1,1,2,3,3,3-hexafluoro-2-(perfluoropropoxy)propoxy)propanoic acid (HFPO-TA), perfluoro-2,5,8-trimethyl-3,6,9-trioxadodecanoic acid (HFPO-TeA), 1-(trifluorovinyloxy)-2-(2-sulfotetrafluoroethoxy)hexafluoropropane (Nafion BP1), 7 H-perfluoro-4-methyl-3,6-dioxa-octanesulfonic acid (Nafion-BP2), perfluoro-(3-oxapentane-1-sulfonic acid) (PF2EOESA), perfluoro-3-methoxypropanoic acid (PFMOPrA) and perfluoro-4-methoxybutanoic acid (PFMOBA) were purchased from SynQuest Laboratories (Alachua, USA). Ammonium perfluorooctanoate (PFOA-NH4), perfluorobutanoic acid (PFBA), perfluorobutanesulfonic acid (PFBS), perfluorodecanoic acid (PFDA), perfluoroheptanoic acid (PFHpA), undecafluorohexanoic acid (PFHxA), perfluorohexanesulfonic acid potassium salt (PFHxS), perfluorononanoic acid (PFNA), perfluorooctanoic acid (PFOA), perfluorooctanesulfonic acid potassium salt (PFOS), perfluoropentanoic acid (PFPeA), pentafluoropropionic acid (PFPrA) and perfluoroundecanoic acid (PFUnA) were purchased from Sigma-Aldrich (Taufkirchen, Germany). 2,2-Difluoro-2-(trifluoromethoxy) acetic acid (PFMOAA) was purchased from Enamine (Frankfurt am Main, Germany). The structures of these PFAS congeners and the CAS numbers can be found in Supplementary Table 1.

It has been reported that some PFECA congeners, e.g., HFPO-DA, are stable in isopropanol, but not in polar, aprotic solvents such as DMSO (Zhang et al. 2022). Thus, isopropanol was chosen as standard solvent to prepare 0.5 M stock solutions for the different PFAS congeners. Four of the 33 PFAS congeners, however, were not sufficiently soluble in isopropanol. Thus, divergent stock solutions were prepared for similar to Nafion-BP2 (0.1 M in isopropanol), PFHxS and PFOS (0.5 M in DMSO), and 8:2 Cl-PFESA (0.05 M in DMSO).

### Cell culture

HepaRG cells were purchased from Biopredic International (St. Gregoire, France) and cell culture was conducted according to the manufacturer’s protocol. HepaRG cells were seeded and cultivated for 28 days in William’s E medium with phenol red (P04-29150, PAN Biotech, Aidenbach, Germany) supplemented with 10% (v/v) fetal bovine serum (FBS) (P40-47100, Lot No. P131102, PAN Biotech), 5 µg/mL insulin (PAN Biotech), 50 µM hydrocortisone hemisuccinate (Sigma-Aldrich, Taufkirchen, Germany), 100 U/mL penicillin and 100 µg/mL streptomycin (Capricorn Scientific, Ebsdorfergrund, Germany) at 37 °C in a humidified atmosphere containing 5% CO_2_. In order to initiate cellular differentiation, 1% (v/v) dimethyl sulfoxide (DMSO) was added to the medium on days 14 and 15, and 1.7% (v/v) DMSO was added to the medium from day 16 to day 28. Medium was changed every 2–3 days during the 28-day cultivation period. Subsequently, the differentiated HepaRG cells were cultivated for two days in serum-free medium as described in Klein et al. (2014) consisting of phenol red-free William’s E medium (P04-29510S1, PAN Biotech, Aidenbach, Germany), 5 µg/mL insulin, 50 µM hydrocortisone hemisuccinate, 100 U/mL penicillin and 100 µg/mL streptomycin, 0.5% (v/v) DMSO, 1% (v/v) Insulin-Transferrin-Selenium 100x solution (Capricorn Scientific, Ebsdorfergrund, Germany), 10 ng/mL human hepatocyte growth factor (100–39 H, Thermo Fisher Scientific, Darmstadt, Germany), 2 ng/mL human epidermal growth factor (AF-100-15, Thermo Fisher Scientific, Darmstadt, Germany), 1 mg/mL bovine serum albumin (BSA) (CP84, Carl Roth, Karlsruhe, Germany). On day 30, cells were treated for 24 h with the different PFAS congeners, by diluting the stock solutions mentioned above with serum-free medium to obtain the required concentrations, thereby adjusting the solvent to 0.1% isopropanol and 0.5% DMSO under all conditions.

### Cytotoxicity

Cytotoxicity of the 33 PFAS congeners was determined by means of the 3-(4,5-dimethylthiazol-2-yl)-2,5-diphenyltetrazolium bromide (MTT) assay. HepaRG cells were seeded with a density of 9.000 cells/100 µL/well in the inner 60 wells of a 96 well plate. The outermost row was filled with 100 µL of a phosphate buffered saline solution. HepaRG cells were cultivated according to the 30-day differentiation procedure described above. On day 30, the medium was removed and the cells were incubated with different PFAS concentrations in serum-free medium for 24 h. Subsequently, MTT-solution (final concentration per well 0.05 mg/mL) was added to the wells, cells were incubated for 1 h at 37 °C in a humidified atmosphere with 5% CO_2_. Subsequently, plates were centrifuged at 300 × g for 5 min, and the incubation solution was replaced by 130 µL desorption-solution (0.7% sodium dodecyl sulfate in isopropanol). After 15 min shaking in the dark (microplate shaker, 600 U/min), absorption was measured at 570 nm (reference wavelength 630 nm), using a microplate reader (Infinite M Flex, Tecan). Three independent biological replicates with three technical replicates were performed. 0.01% Triton-X 100 served as positive control (PC). Cellular viability of treated cells was calculated relative to the untreated negative (solvent) control (SC), which contained 0.1% (v/v) isopropanol and 0.5% DMSO.

### RNA preparation

HepaRG cells were seeded with a density of 2*10^5^ cells/2 mL/well in a 6 well plate. HepaRG cells were cultivated according to the 30-day differentiation procedure described above. On day 30, the medium was removed and the cells were incubated with different PFAS concentrations in serum-free medium for 24 h. Subsequently, cells were visually checked under the microscope. Cell lysis and extraction of total RNA was done according to the protocol of the RNeasy Mini Kit (catalogue number 74104, Qiagen, Venlo, Netherlands). Three independent biological replicates with one technical replicate were performed. Cell lysates and isolated RNA were stored at − 80 °C.

### RNA library preparation and sequencing

RNA samples were initially quantified using the RNA Assay Kit (Thermo Fisher Scientific, Waltham, Massachusetts, USA) on a Qubit^®^ 4.0 Fluorometer. Sample integrity was assessed using the Fragment Analyzer with the High Sensitivity RNA Analysis Kit (Agilent Technologies, Santa Clara, California, USA). RNA quality was evaluated based on the RNA Quality Number (RQN), and the value was higher than 7 for each sample. Library preparation was performed using the SMART-Seq mRNA LP Kit (Takara Bio Inc., San Jose, CA, USA). First-strand cDNA synthesis from total RNA was carried out using the 3′ SMART-Seq CDS Primer II A, with template switching at the 5′ end enabled by the SMART-Seq v4 Oligonucleotide. The resulting cDNA was amplified using PCR Primer II A, targeting sequences introduced during cDNA synthesis. Amplified cDNA was purified with NucleoMag NGS Clean-up and Size Select beads (Takara Bio Inc.) and quantified using the DNA HS Assay Kit on a Qubit^®^ 4.0 Fluorometer (Thermo Fisher Scientific, Waltham, MA, USA). Purified cDNA was diluted to 2 ng/µL and subjected to 14 cycles of PCR to generate sequencing libraries, during which dual indices were incorporated for sample multiplexing. Library size distribution was evaluated using the Fragment Analyzer with the High Sensitivity Small Fragment Analysis Kit (Agilent Technologies, Santa Clara, California, USA). Amplified libraries were pooled, purified, and quantified using the KAPA Library Quantification Kit (Roche Diagnostics), based on quantitative PCR measurements and average library size. The pooled libraries were loaded onto an Illumina NovaSeq and NextSeq 550 flow cells (Illumina, San Diego, California, USA) and sequenced in a 2 × 75 bp paired-end format at the Polo GGB sequencing facility (Polo d’Innovazione di Genomica, Genetica e Biologia Srl, Siena, Italy).

### Processing of RNAseq data

Demultiplexing and conversion to FASTQ files was accomplished using Illumina bcl2fastq version 2.20. The quality of raw data was assessed by FastQC version 0.12.1 (Andrews 2010) and multiQC version 1.21 (Ewels et al. 2016). Quality filtering was then performed using Trimmomatic version 0.36 to remove adapters and low-quality reads. Then reads were mapped to the human reference genome “GRCh38” and Ensembl 110 annotations using kallisto version 0.50.1 with default parameters. After removing the genes with zero counts across all samples, the retained 34,904 genes were analysed in R version 4.4.1 (R Core Team 2024). Removal of batch effects due to technical differences was performed by the “ComBat_seq” function from the R package sva, version 3.52.0. Differential gene expression (DGE) analysis was done using the R package DESeq2, version 1.44.0 (Love et al. 2014) by using default settings for estimation of size factors and dispersion. Negative Binomial GLM fitting and Wald statistics were applied to test for DGE between each treatment and control conditions, respectively. False discovery rate (FDR) was used to control for multiple testing (Benjamini and Hochberg 1995), with the “independent filtering” being performed by default. Only genes with p_adj_ < 0.05 and |log_2_FC|> 0.5 were identified as DEGs and included in the further analyses. Heatmaps were generated by the R package ComplexHeatmap version 2.20.0 (Gu et al. 2016).

### Ingenuity pathway analysis

Processed RNAseq datasets were uploaded to IPA software (Qiagen; spring release, Q1 2025) using ENSG numbers as identifiers. Data were analysed using the following cutoff criteria: |log_2_FC| > 0.5 and p_adj_ < 0.05. In addition, the analyses were restricted to species “human” and to tissues and cell lines “liver”, “hepatocytes” and “hepatoma cell lines”. Finally, only direct relationships were considered for network and upstream regulator analysis. Comparison analyses were conducted for the three concentration categories “high”, “medium” and “low”. Since the IPA software only allows comparison analyses of maximal 20 different data sets, one comparison analysis was conducted for all PFCA and PFSA congeners (15 congeners), and a second comparison analysis included all congeners of the PFECA and PFESA subgroups (18 congeners). Subsequently, the results of the two analyses were exported from IPA and assembled in R to obtain one comparison analysis for all 33 PFAS congeners.

## Results

### Cytotoxicity and selection of PFAS concentrations

**Fig. 1:**
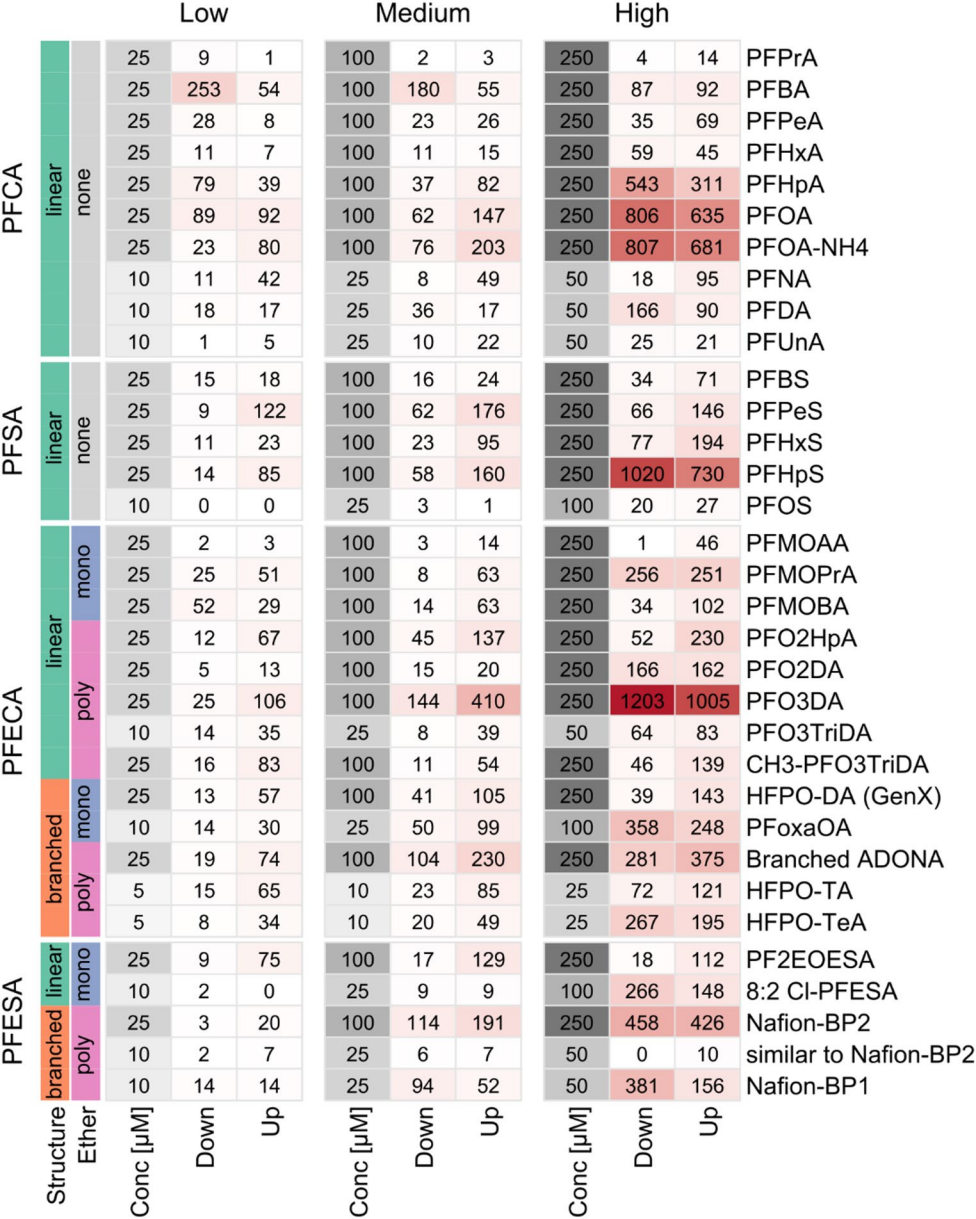
Summary of results from DGE analysis for 33 PFAS in HepaRG cells. The substances (rows) are split by their subgroups (PFCA, PFSA, PFECA and PFESA) and color coding on the left indicates mono- and polyether PFAS as well as linear and branched structures. The columns are split in blocks by the three concentration categories “low”, “medium” and “high”. Within each block the respective concentration [µM] is color coded in grey, while the number of up- and down-regulated genes is indicated by the red color gradient

For each PFAS, three non-cytotoxic concentrations were selected for the HepaRG treatments for the transcriptomic study. The MTT assay, which determines the activity of mitochondrial dehydrogenases, is frequently used as a measure for cellular viability and was therefore used to evaluate cytotoxic effects of the 33 PFAS congeners in differentiated HepaRG cells. For each congener, an initial MTT screening was conducted at a broader concentration range (data not shown). Many of the 33 PFAS were not cytotoxic at concentrations of 500 µM or higher, however, 250 µM was selected as the maximal test concentration for the entire project, referred to as “high” concentration in the following. Additional MTT assays were conducted at a smaller concentration range to confirm that the respective selected “high” concentration was not cytotoxic in HepaRG cells (Supplementary Fig. 1). Two additional test concentrations were selected for each PFAS, referred to as “medium” and “low” concentrations in the following. Overall, the selected test concentrations cover a range between 5 µM and 250 µM. All PFAS were tested at a concentration of 25 µM, however, this was the “low” concentration in most cases. 22 of the 33 congeners were tested at 250 µM, and 25 of the 33 congeners were tested at 100 µM. For each PFAS, the concentrations used for HepaRG treatment are depicted in Fig. 1. It has to be noted that the subsequent comparison analyses that were conducted to compare the transcriptomic data of the 33 PFAS are based on the “high”, “medium” and “low” classification and not on the numerical PFAS concentrations.

### Experimental workflow

Differentiated HepaRG cells were incubated for 24 h with the three selected concentrations of the 33 PFAS congeners (Fig. 1). Three independent replicates derived from different passages were generated per treatment condition. In total, 305 samples were prepared, including 8 untreated control samples. Total RNA was prepared from these samples, checked for RNA integrity, and subjected to whole transcriptome analysis by using the Illumina NovaSeq and NextSeq 550 platforms. Sequence alignment was conducted with kallisto, and after filtering and removal of batch effects, DGE analysis was conducted by using a cutoff of |log_2_FC| > 0.5 and p_adj_ < 0.05.

### DGE analysis

The results of the DGE analyses are summarized in Fig. 1. Concentration-dependent increases of the numbers of deregulated genes were observed for most PFAS congeners. Some PFAS, e.g., PFOA, PFHpS and PFO3DA, induced strong transcriptomic alterations at least at the respective “high” test concentration, whereas other PFAS, e.g., the short-chain congeners PFPrA and PFMOAA as well as PFUnA, PFOS and similar to Nafion-BP2, hardly affected gene expression in HepaRG cells. Notably, many treatment conditions induced only marginal alterations in gene expression. This accounts in particular for the “low” test concentrations. By selecting an arbitrary threshold of 100 DEGs being the minimal number for a considerable PFAS-mediated effect on gene expression, this value was reached for only 6 of the 33 PFAS at the “low” test concentration, for 16 of the 33 PFAS at the “medium” test concentration and for 28 of the 33 PFAS at the “high” test concentration. In order to compare the PFAS-mediated effects at the transcriptional level for the entire set of 33 PFAS, subsequent pathway analysis was conducted for all PFAS and for all test concentrations in spite of the low number of DEGs for many treatment conditions.

### IPA analysis

**Fig. 2:**
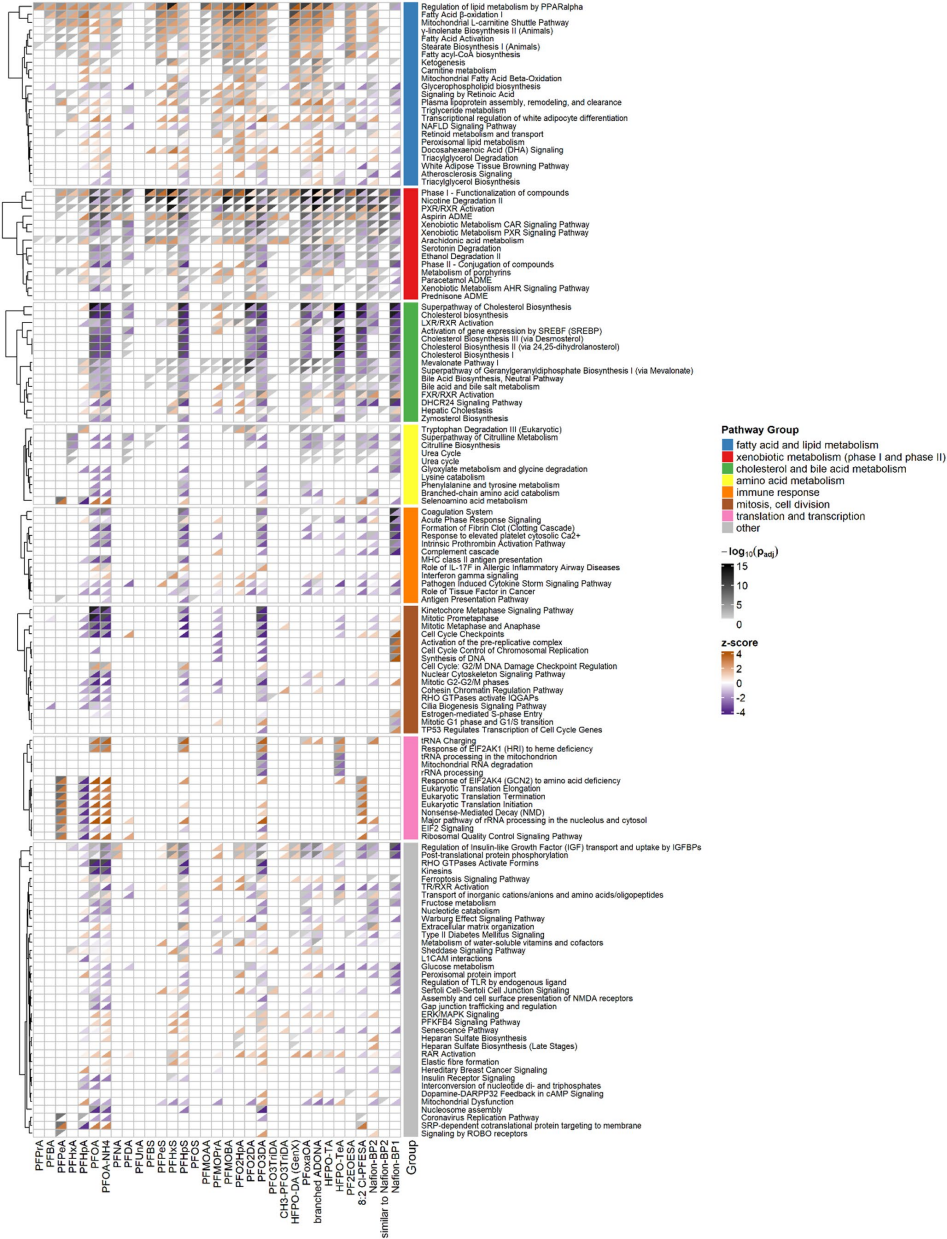
Comparison analysis of the IPA canonical pathways based on the data from the “high” concentration of the 33 PFAS. A canonical pathway is displayed when the following criteria were fulfilled: for the entire datasets of all 33 PFAS, at least one |z-score| > 1 and at least one p_adj_ < 0.05. The pathways were sorted according to pathway groups representing specific biological functions as indicated in the figure legend. Within each pathway group, the pathways were sorted according to p_adj_. When p_adj_ < 0.05, it is presented in light-gray to black with increasing -log_10_(p_adj_)-values at the upper left triangles of the heatmap tiles. At the lower right triangles of the heatmap tiles, the IPA z-scores are given in orange (meaning activation of the respective pathway) and violet (meaning inhibition of the respective pathway)

**Fig. 3:**
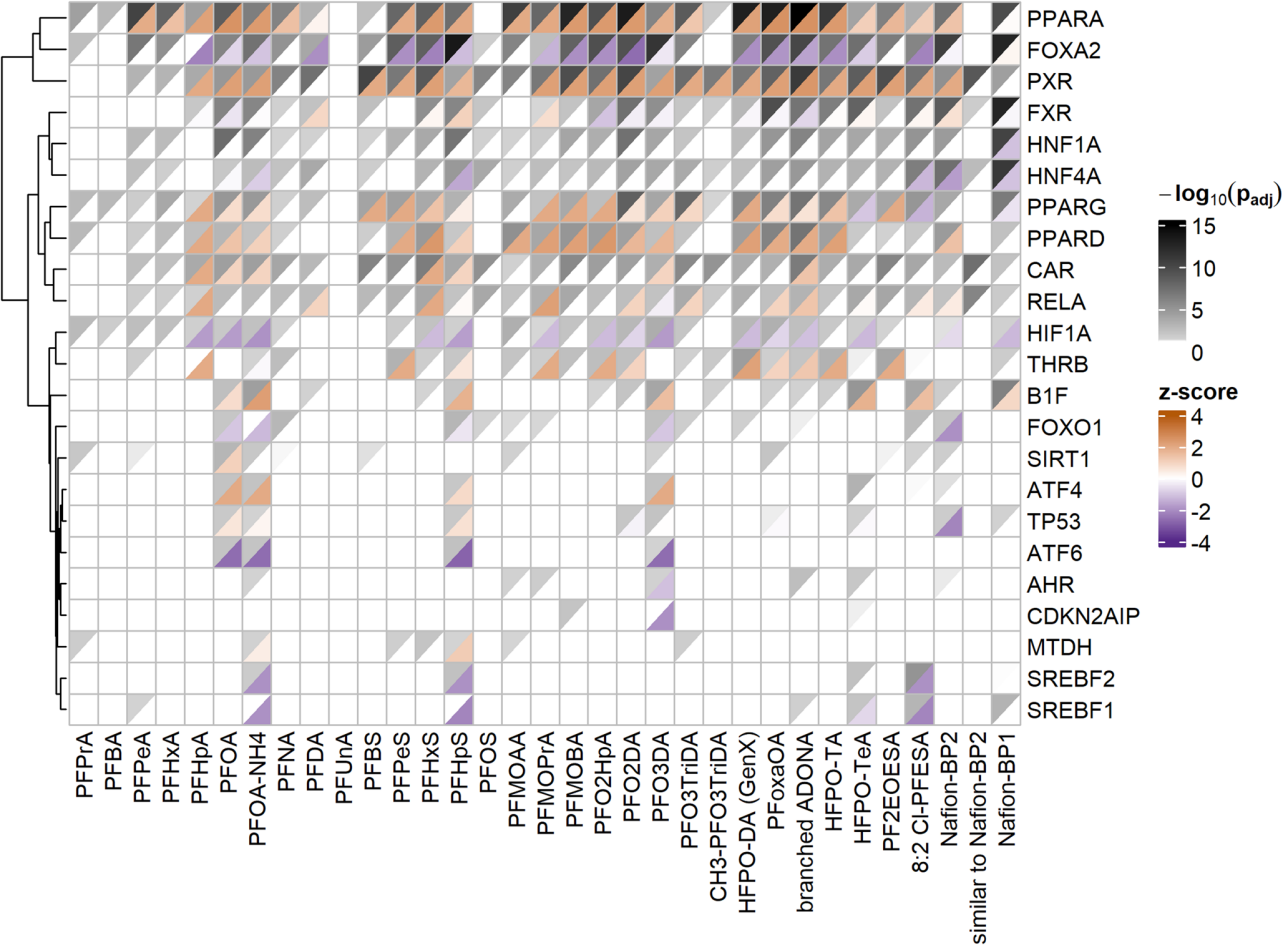
Comparison analysis of the IPA upstream regulators based on the data from the “high” concentration of the 33 PFAS. An upstream regulator is displayed when the following criteria were fulfilled: for the entire datasets of all 33 PFAS, at least one |z-score| > 1 and at least one p_adj_ < 0.05. The upstream regulators were sorted according to p_adj_. When p_adj_ < 0.05, it is presented in light-gray to black with increasing -log_10_(p_adj_)-values at the upper left triangles of the heatmap tiles. At the lower right triangles of the heatmap tiles, the IPA z-scores are given in orange (meaning activation of the respective upstream regulator) and violet (meaning inhibition of the respective upstream regulator)

**Fig. 4:**
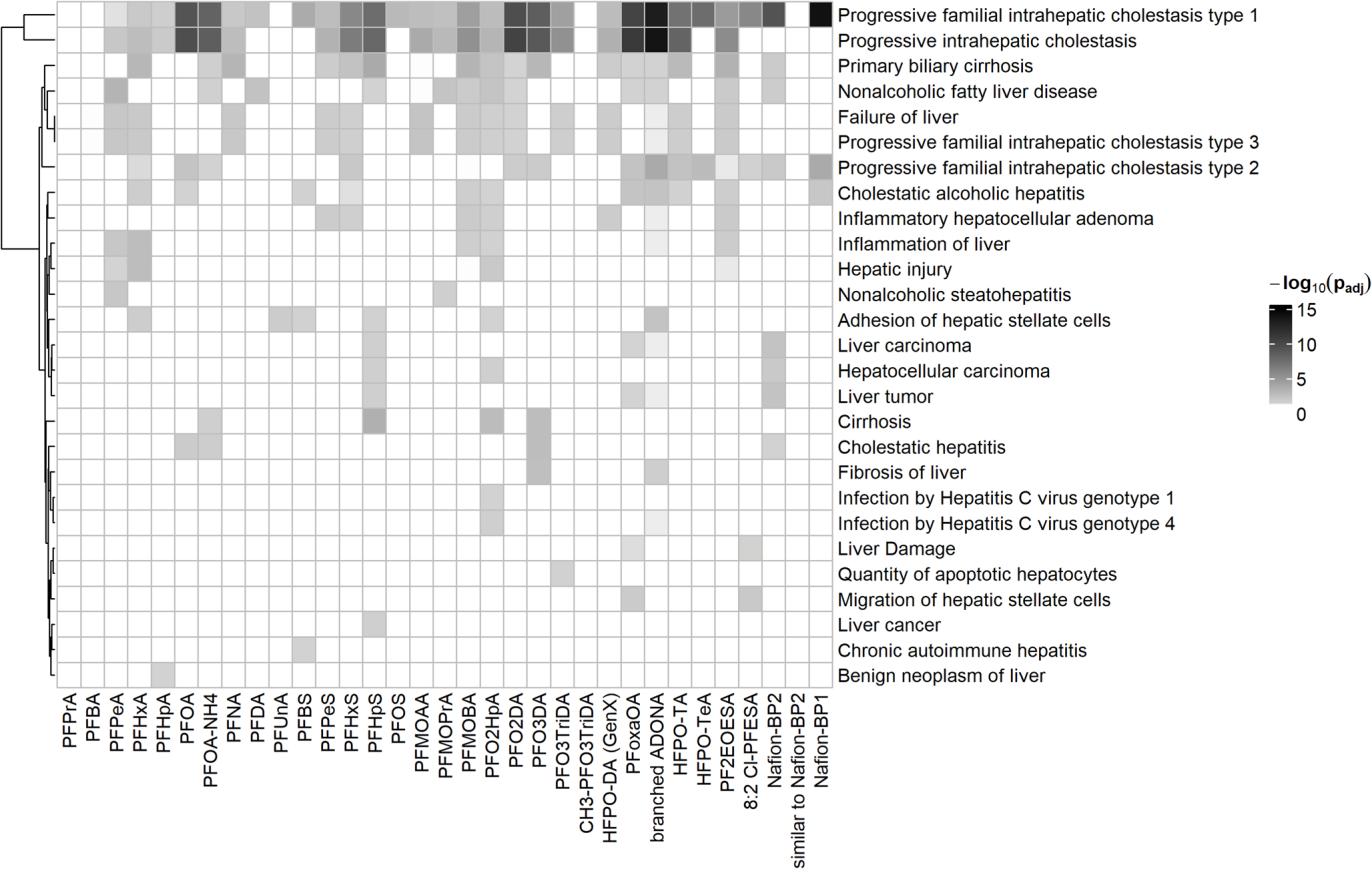
Comparison analysis of the IPA downstream effects (tox functions) based on the data from the “high” concentration of the 33 PFAS. A downstream effect is displayed when at least one p_adj_ < 0.05 was calculated by IPA for the entire datasets of all 33 PFAS. The downstream effects were sorted according to p_adj_. When p_adj_ < 0.05, it is presented in light-gray to black with increasing − log_10_(p_adj_)-values

IPA analysis was conducted by using cutoff criteria of |log_2_FC| > 0.5 and a p_adj_ < 0.05. In addition, the analyses were restricted to species “human”, to organ “liver” and to cell types “hepatocytes” and “hepatocarcinoma cell lines” to ensure that our own experimental data obtained with HepaRG cells were only compared with experimental data from the various IPA sources that have been obtained with human liver, human primary hepatocytes or human hepatocarcinoma cell lines. Moreover, only direct interactions were allowed and data related to indirect interactions were excluded. Finally, comparison analyses were conducted for the three concentration categories “high”, “medium” and “low”. The comparison analysis for the “high” concentration group is presented in Fig. 2 for the canonical pathways, in Fig. 3 for the upstream regulators, and in Fig. 4 for downstream effects (tox functions). The corresponding comparison analyses for the “medium” and “low” concentration categories are given in the Supplementary Figs. 2–7.

### Canonical pathways; overall data interpretation

As expected, the impact of the different PFAS on the various canonical pathways correlated with the number of DEGs. The strongest effects were observed for PFOA, PFOA-NH4, PFHpS and PFO3DA which also displayed the highest numbers of DEGs (Fig. 1). Notably, the impact of PFOA and PFOA-NH4 on the canonical pathways was nearly identical, regarding both the significance and the z-scores. Thus, it was obviously irrelevant whether PFOA was applied as the free acid or as the ammonium salt to this cell culture experiment. Strong effects on canonical pathways were also observed for some PFECA and PFESA congeners, e.g., PFoxaOA, HFPO-TeA, 8:2 Cl-PFESA and Nafion-BP1. Only minor effects were observed for those PFAS that in general had minor effects on gene expression in HepaRG cells as reflected by the small number of DEGs (Fig. 1); these were PFPrA, PFBA, PFUnA, PFOS, PFO3TriDA, CH3-PFO3TriDa and similar to Nafion-BP2.

Notably, several canonical pathways were affected by nearly all PFAS tested in this study, pointing to molecular effects shared by all members of the PFCA, PFSA, PFECA and PFESA subgroups. These common pathways are associated in particular with lipid metabolism and xenobiotic metabolism. Additional pathways were affected by a smaller number and not by the entire set of the 33 PFAS used in this study. These pathways are related to cholesterol and bile acid metabolism, amino acid metabolism, cell division, translation, transcription, and immune functions. The various canonical pathways have been sorted into different pathway groups that are highlighted by color in Fig. 2 and that are described in more detail in the following.

### Fatty acid and lipid metabolism

A number of canonical pathways associated with fatty acid uptake, activation, transport, metabolism and storage were activated by all PFAS except for PFUnA, PFOS, CH3-PFO3TriDA and similar to Nafion-BP2. As presented in Fig. 2, pathways such as “fatty acid β-oxidation I”, “mitochondrial L-carnitine shuttle pathway”, “fatty acid activation”, or “stearate biosynthesis I (animals)” were clearly activated as indicated by the positive z-scores (orange color). “Regulation of lipid metabolism by PPARalpha” was the top affected, lipid-associated canonical pathway, pointing to an important role of PPARα in PFAS-mediated alteration of lipid metabolism. The impact of the 33 PFAS on expression of the genes associated with this specific canonical pathway is depicted in Supplementary Figs. 8–10. Indeed, gene expression of some PPARα target genes such as *PLIN2*, *FABP1*, *CPT1A* and *ACOX1* was clearly upregulated in HepaRG cells upon incubation with most PFAS congeners at all three concentration levels. Moreover, as predicted by IPA, PPARα was the top upstream regulator activated by almost all PFAS (Fig. 3). In addition, PPARγ and PPARδ, nuclear receptors that are like PPARα involved in the regulation of lipid metabolism, were also activated by most PFAS (Fig. 3). As an example, PPARγ is the central transcription factor of the canonical pathway “transcriptional regulation of white adipocyte differentiation”. Thus, the strong impact of PFAS on fatty acid and lipid metabolism can largely be explained by a PFAS-mediated activation of all three PPAR subtypes.

Notably, PFAS-mediated activation of fatty acid and lipid metabolism was observed not only for the “high” concentration category (Fig. 2), but also for the “medium” and partly for the “low” concentration categories (Supplementary Figs. 2 and 3), indicating that all PFAS affect these pathways at a broader concentration range.

### Xenobiotic metabolism

In addition to lipid metabolism, nearly all PFAS largely affected canonical pathways related to xenobiotic metabolism. As for the lipid metabolism, this was again observed for the broad concentration range of the “high”, the “medium”, and partly the “low” concentration categories. PFAS affected xenobiotic metabolism pathways that are based on the gene expression of both phase I and phase II enzymes. Alteration of CYP and/or ADH gene expression (phase I) is represented by pathways such as “phase I – functionalization of compounds”, “nicotine degradation II”, “aspirin ADME” and “ethanol degradation”, and alteration of UGT and/or GST gene expression (phase II) is represented by pathways such as “phase II – conjugation of compounds”, “serotonin degradation”, “metabolism of porphyrins” and “paracetamol ADME” (Fig. 2). Obviously, PXR and CAR are the central upstream regulators of these xenobiotic pathways that are activated by all PFAS except for PFPrA, PFBA and PFUnA (Fig. 3). Interestingly, PFAS-mediated activation of these two nuclear receptors is not fully reflected in the canonical pathways. Regarding the z-scores, there is a trend towards activation of the pathway “PXR/RXR activation”, whereas the z-scores point into the direction of an inhibition of the pathways “xenobiotic metabolism CAR signaling pathway” and “xenobiotic metabolism PXR signaling pathway”. Thus, it has to be noted that PFAS strongly affect PXR- and CAR-regulated gene expression in HepaRG cells, however, the direction of regulation (activation or inhibition) is not that clear compared to the clear PPAR-mediated activation of lipid metabolism. The uncertainties regarding the direction of regulation can be explained by the fact that many PXR- and CAR-regulated genes were upregulated upon PFAS-treatment of HepaRG cells, whereas other PXR- and CAR-dependent genes were downregulated at the same time. This is illustrated in Supplementary Figs. 11–13, showing the impact of the 33 PFAS on expression of the genes associated with the canonical pathway “phase I – functionalization of compounds”. Classic PXR and CAR target genes such as *CYP3A4* and *CYP2B6* were strongly upregulated whereas other CYP genes (e.g., *CYP1A1* and *CYP2E1*) were downregulated upon PFAS treatment of HepaRG cells. Thus, PFAS-mediated alteration of xenobiotic metabolism may not exclusively rely on PXR and/or CAR activation, but may also involve additional mechanisms.

### Cholesterol and bile acid biosynthesis

**Fig. 5:**
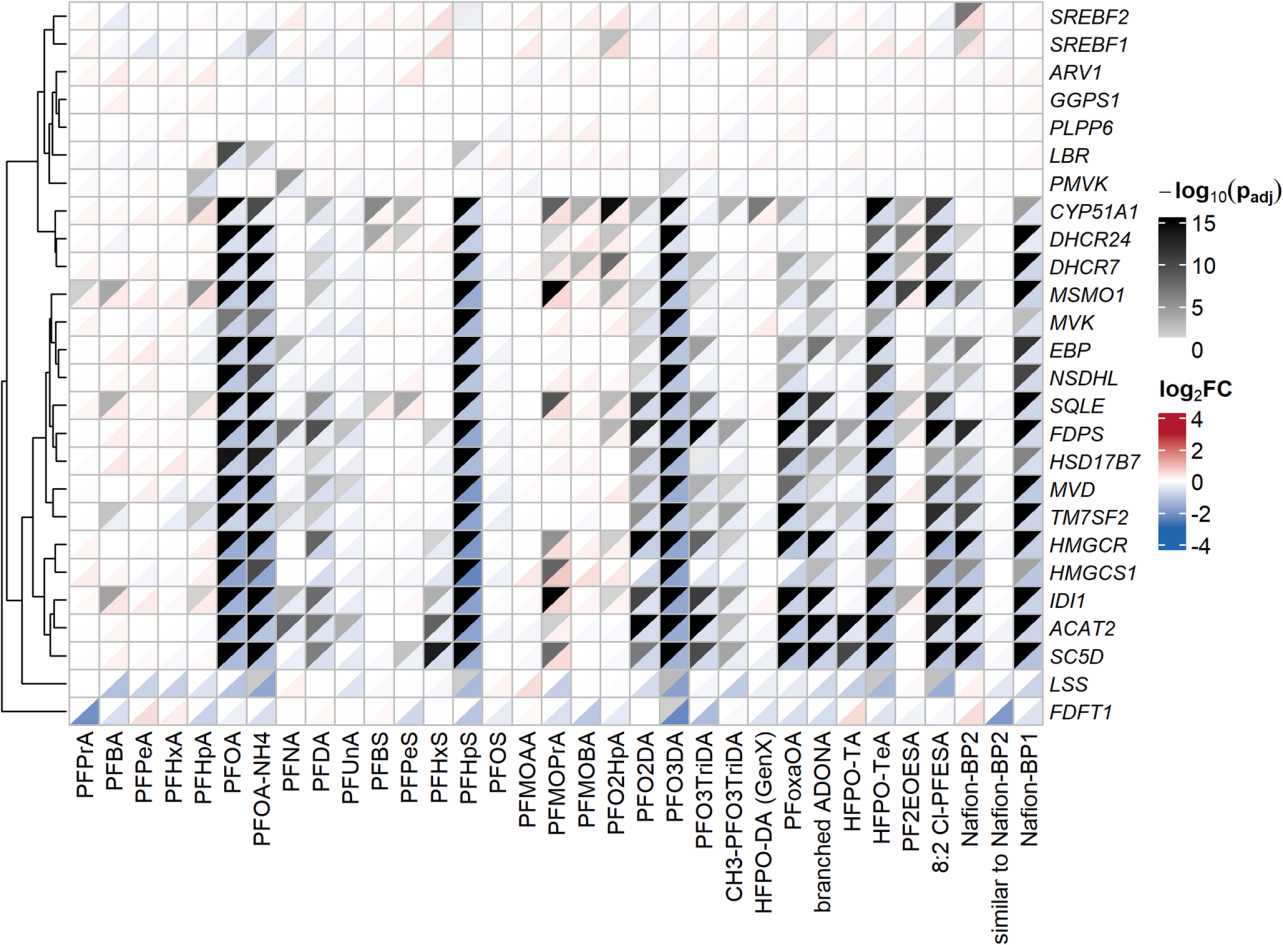
Comparison analysis of the genes of the IPA canonical pathway “cholesterol biosynthesis” based on the data from the “high” concentration of the 33 PFAS. All genes that belong to the IPA-pathway “cholesterol biosynthesis” are displayed. The genes were sorted according to log_2_FC from positive values at the top to negative values at the bottom. When p_adj_ < 0.05, it is presented in light-gray to black with increasing − log_10_(p_adj_)-values at the upper left triangles of the heatmap tiles. At the lower right triangles of the heatmap tiles, log_2_FC are given in red (meaning upregulation of expression of the respective gene) and blue (meaning downregulation of expression of the respective gene)

**Fig. 6:**
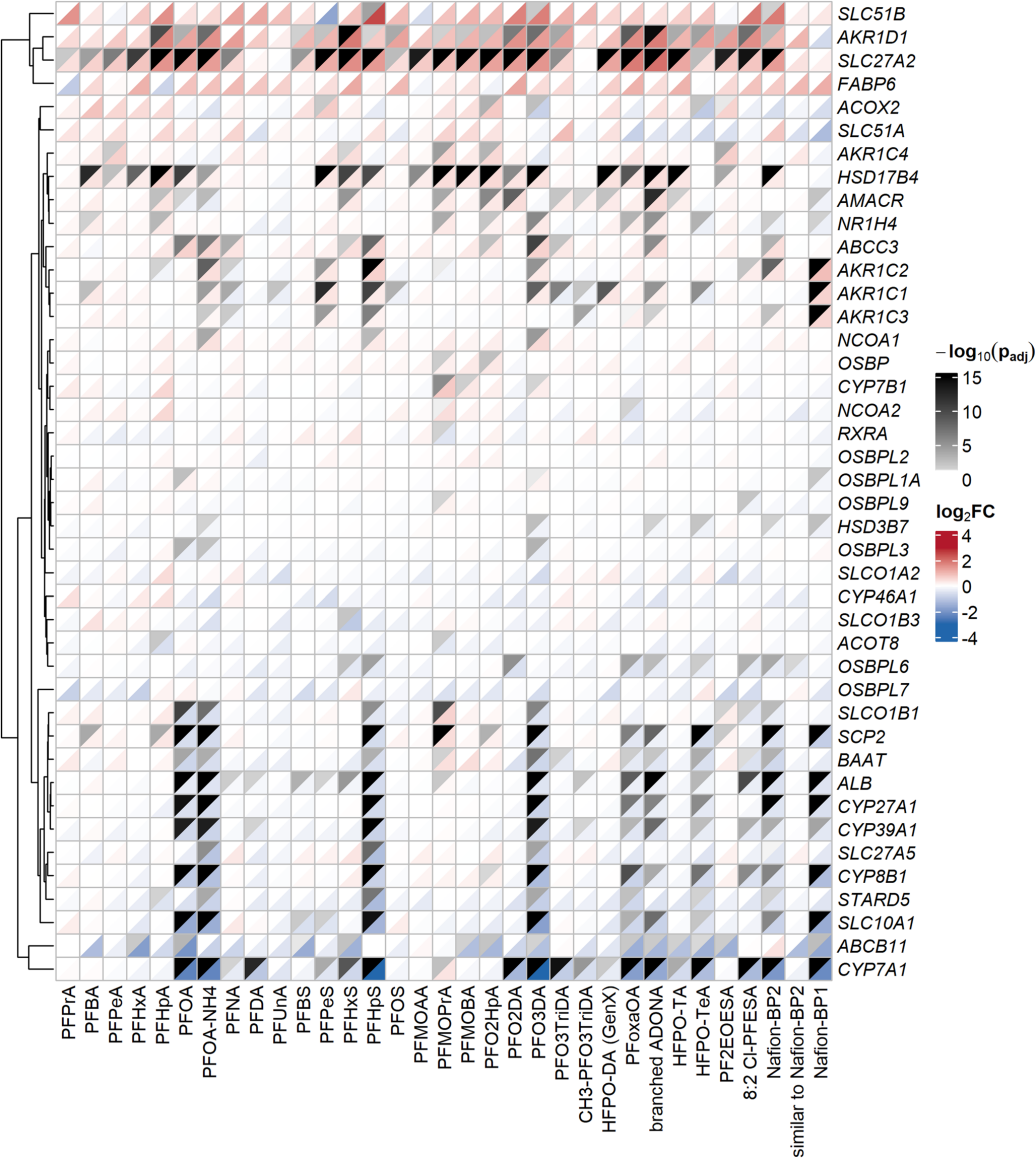
Comparison analysis of the genes of the IPA canonical pathway “bile acid and bile salt metabolism” based on the data from the “high” concentration of the 33 PFAS. All genes that belong to the IPA-pathway “bile acid and bile salt metabolism” are displayed. The genes were sorted according to log_2_FC from positive values at the top to negative values at the bottom. When p_adj_ < 0.05, it is presented in light-gray to black with increasing − log_10_(p_adj_)-values at the upper left triangles of the heatmap tiles. At the lower right triangles of the heatmap tiles, log_2_FC are given in red (meaning upregulation of expression of the respective gene) and blue (meaning downregulation of expression of the respective gene)

Some of the tested PFAS strongly inhibited canonical pathways associated with cholesterol and bile acid biosynthesis (Fig. 2). This accounts for those PFAS with a strong impact on the HepaRG transcriptome, indicated by a high number of DEGs. As mentioned above, these are PFOA, PFOA-NH4, PFHpS and PFO3DA. In addition, PFoxaOA, HFPO-TeA, 8:2 Cl-PFESA and Nafion-BP1 strongly inhibited these pathways related to cholesterol and bile acid biosynthesis. This is exemplified for the canonical pathways “cholesterol biosynthesis” (Fig. 5 and Supplementary Figs. 14–15) and “bile acid and bile salt metabolism” (Fig. 6 and Supplementary Figs. 16–17), depicting the impact of the 33 PFAS on the expression of the genes associated with these pathways in HepaRG cells. Some PFAS strongly downregulated nearly all genes whose products are involved in the cholesterol biosynthesis pathway (Fig. 5), pointing to a common regulation of these genes. Gene expression of *SREBF1* and *SREBF2*, central regulators of cholesterol biosynthesis, is hardly affected by PFAS (Fig. 5), however, IPA predicted these two transcription factors to be inhibited in their regulatory function by some PFAS (Fig. 3). Additional upstream regulators involved in the regulation of cholesterol biosynthesis were predicted to be activated (B1F) or inhibited (ATF6) by some PFAS (Fig. 3). In the case of bile acid metabolism, *CYP7A1* gene expression was massively downregulated by many PFAS (Fig. 6). Moreover, expression of additional genes encoding enzymes involved in bile acid biosynthesis (e.g., *CYP8B1*, *CYP27A1*, *AKR1D1*) as well as genes encoding membrane transporters involved in bile acid excretion (*ABCB11*, *SLC51B*, *SLC51A*) was affected by a number of PFAS (Fig. 6).

Notably, both the canonical pathways and the corresponding upstream regulators involved in cholesterol and bile acid biosynthesis were affected only at the “high” concentrations of the respective PFAS (Figs. 2 and 3), but there was no significant impact at the “medium” or “low” concentrations (Supplementary Figs. 2–5), which is in contrast to the impact of PFAS on lipid metabolism and xenobiotic metabolism described above. In view of the fact that the high concentration of 250 µM was close to the cytotoxicity threshold for, e.g., PFOA, PFOA-NH4, PFHpS and PFO3DA, the strong impact of these PFAS on cholesterol and bile acid biosynthesis can be interpreted as massive cellular metabolic and regulatory disturbances that seem to occur at concentrations close to those that induce cell death. Although these effects were only observed for some of the tested PFAS and only at near-cytotoxic concentrations, it cannot be excluded that also other PFAS may inhibit cholesterol and bile acid biosynthesis when tested at higher concentrations.

### Additional canonical pathways

In addition to the pathways associated with cholesterol and bile acid biosynthesis, the PFAS with the strong impact on the HepaRG transcriptome (high number of DEGs) also affected some canonical pathways associated with amino acid metabolism, cell division, general transcription and translation, and certain immune-related responses (Fig. 2). Pathways related to these global cellular functions were either activated or inhibited by certain PFAS at the respective “high” concentration, but not at the “medium” and “low” concentrations (Supplementary Figs. 2 and 3), again pointing to massive disturbances in cellular signaling and metabolism that seem to occur at concentrations close to those that induce cell death. The impact of the PFAS on these various cellular functions is less pronounced in terms of low significance and small z-scores compared to the strong impact on cholesterol and bile acid biosynthesis described above. Overall, no clear picture arises from these various pre-cytotoxic effects induced by a few PFAS close to the respective cytotoxicity threshold, and interpretation of these data should be handled with care.

### Additional upstream regulators

IPA analysis revealed that most PFAS inhibited FOXA2 which is a liver-specific transcription factor involved in hepatocyte differentiation. Moreover, the hepatocyte-specific global regulators HNF4A and HNF1A were predicted by IPA to be significantly affected by most PFAS with a trend to inhibition (Fig. 3). In addition, HIF1A, the alpha subunit of the HIF-1 transcription factor which is a master regulator of cellular response to hypoxia, was predicted to be inhibited by most PFAS. All four transcription factors are involved in the regulation of central cellular functions such as proliferation, differentiation, energy metabolism and metabolic homoeostasis, indicating that PFAS were globally impacting cellular viability of HepaRG cells. PFAS-mediated inhibition of these four central regulators was predicted for the “high”, “medium” and partially also at the “low” concentration categories (Fig. 3, Supplementary Figs. 4 and 5), indicating that these central cellular functions are affected by PFAS at a broader concentration range. This also holds true for THRB, a nuclear receptor involved in regulation of thyroid metabolism, that was predicted to be activated by many PFAS of the different subgroups at “high”, “medium” and “low” concentration, pointing to an impact of PFAS on the thyroid endocrine system. Finally, the NF-κB subunit RELA was predicted to be activated by many PFAS at the broader concentration range, linking the PFAS-mediated effects also to NF-κB signaling.

In addition to these various transcription factors that were affected by nearly all PFAS, some upstream regulators such as TP53, AHR and ATF4 were affected by a few PFAS used in this study (Fig. 3), however, these findings were not very robust, and no clear picture arises from these observations. Of note, these interactions with some additional regulators were predominantly restricted to members of the PFECA and PFESA subgroups, indicating that the novel PFAS may affect some additional regulators that are not targeted by legacy PFAS of the PFCA and PFSA subgroups.

### Downstream effects (tox functions)

Regarding downstream effects, the IPA software predicted a strong association between the molecular effects induced by most PFAS used in this study and some liver-specific adverse outcomes in vivo, in particular “progressive intrahepatic cholestasis” and “progressive familial intrahepatic cholestasis type 1” (Fig. 4). These associations were predicted for the “high”, the “medium”, and partly for the “low” concentration categories (Supplementary Figs. 6 and 7). Although with less robust significance, additional diseases related to disturbances in bile acid metabolism (e.g., progressive familial intrahepatic cholestasis types 2 and 3, primary biliary cirrhosis) and lipid metabolism (e.g., nonalcoholic fatty liver disease) were predicted as well as diseases associated with inflammation (e.g., inflammatory hepatocellular adenoma, inflammation of liver), finally including even cirrhosis, hepatocellular carcinoma or liver cancer as terminal adverse outcomes related to the molecular effects induced by some PFAS.

The predictions of potential downstream effects are based on indirect associations with unknown causality and should therefore be handled with care. There are numerous reports that, e.g., certain liver diseases are associated with an alteration of hepatocyte gene expression. However, it is not clear whether the altered gene expression is causative for the development of the disease, or whether it is the other way around, i.e., the organ tries to compensate for the metabolic disorder causative for the disease by an altered gene expression. Furthermore, IPA considers a second type of association based on reports on the occurrence of gene mutations in relation to certain diseases. However, the fact that certain diseases are sometimes associated with mutations in specific genes, and that these mutations may probably lead to an altered function of the corresponding gene product, does not immediately lead to the conclusion that the mutation is causative for the disease.

Taken together, the IPA predictions rely on indirect associations with unknown causality and should be taken with care, however, it has to be noted that IPA predicts a strong association between the in vitro PFAS-mediated alterations of gene expression and adverse effects in vivo, mainly related to intrahepatic cholestasis, a disease that develops in the course of continuous imbalance in bile acid biosynthesis and excretion.

## Discussion

Animal and human epidemiological studies have shown that exposure to several legacy PFAS, including PFOA and PFOS, is associated with adverse effects on the immune and the endocrine system, and liver. The underlying molecular mechanisms, however, are still not fully understood. Omics approaches, and transcriptomics in particular, are widely used as hypothesis-free tools to identify affected molecular pathways and to generate mechanistic insight into toxicant action. Numerous transcriptomic studies have investigated PFAS-induced effects across species (including human, mouse, fish, and other environmental species) and across tissues (Attema et al. 2022; Dasgupta et al. 2020; Feng et al. 2022; Khan et al. 2021; Pfohl et al. 2021; Rowan-Carroll et al. 2021). Many of these studies focus mainly on PFOS- and/or PFOA, with some also including additional PFCA and PFSA congeners. A recent study has combined and re-evaluated the results of a number of transcriptomic studies and concluded that PFAS induce broadly comparable transcriptomic changes across species and tissues, suggesting that conserved ancient molecular targets are affected by PFAS (Beccacece et al. 2023). These conserved responses were linked primarily to pathways related to immunity, endocrine signaling, and lipid metabolism.

With a specific focus on liver toxicity, several rodent studies have identified PPARα activation as a key molecular initiating event in PFAS hepatotoxicity. Recently, Robarts et al. (2024) re-analyzed transcriptomic datasets from PFAS-exposed mouse liver, including legacy PFAS (PFOA, PFOS, PFNA, PFHxS) and several alternative PFAS (e.g., HFPO-DA, HFPO-TA, Nafion-BP2). Their analysis showed that, with the exception of Nafion-BP2, the tested PFAS consistently stimulated PPARα-regulated lipid metabolism. In addition, the transcription factors CAR, NRF2 and SREBP1/2 were activated by most PFAS, whereas STAT5b was inhibited. Overall, the authors concluded that legacy and alternative PFAS induce similar molecular responses in mouse liver.

Several transcriptomic studies have been conducted with human hepatocarcinoma cell lines or primary human hepatocytes, most of them with a limited number of legacy PFAS. In HepG2 cells, PFOS, PFOA and PFHxA affected several pathways associated with fatty acid and lipid metabolism, glucose metabolism, and general cell stress (Carberry et al. 2025). In HepaRG cells, PFOS, PFOA and PFNA activated fatty acid and lipid metabolism regulated by PPARα, and inhibited gene expression of genes associated with cholesterol biosynthesis. Alterations in gene expression furthermore pointed to an induction of endoplasmic reticulum stress by these three legacy PFAS (Louisse et al. 2020, 2023). In primary human hepatocytes, HFPO-DA (GenX) altered gene expression of genes involved in lipid, monocarboxylic acid, and ketone metabolism at a concentration range between 0.1 and 100 µM. PPARα, VEGF, STAT3 and SMAD4 signaling was induced at 100 µM HFPO-DA (Robarts et al. 2022). Taken together, PFAS-mediated activation of PPARα-regulated gene expression was a recurrent theme in all these studies conducted with human-derived hepatocytes.

The most comprehensive human hepatocyte-based PFAS transcriptomic study so far, published by Addicks et al. (2025), used human liver spheroids derived from primary human hepatocytes and emphasized that PFAS do not induce identical pathway profiles, and this is partly consistent with our data. However, in our HepaRG dataset the dominant pattern across the entire panel of 33 PFAS remained a broadly comparable pathway-level response, with the clearest shared signal in lipid and xenobiotic metabolism, whereas divergence became more visible mainly in additional pathways emerging at higher, near-cytotoxic concentrations. In this context, the main novelty of the present study is that the transcriptomic response pattern across the 33 PFAS congeners can be viewed as a two-layer response. A first layer, observed for most compounds, comprises a broadly shared core response dominated by lipid metabolism and xenobiotic metabolism. A second layer comprises additional pathway changes (including cholesterol/bile acid metabolism, amino acid metabolism, cell division, translation/transcription and immune-related functions) that were only observed for a subset of PFAS and mainly at the respective “high” concentrations, i.e., close to the cytotoxicity threshold.

An important difference between the Addicks study and our own study is the PFAS panel composition: There was only one PFECA congener in the Addicks study, namely HFPO-DA (GenX), that was also included in our PFAS selection. In this regard, the selection of PFAS congeners of our own study with a focus on (poly)-ether PFAS complements the Addicks study and enlarges the available information on PFAS-induced transcriptomic alterations in human hepatocytes to a number of at least 44 PFAS congeners in total. In this context, to the best of our knowledge, our own study for the first time provides transcriptomic data for at least 13 PFAS congeners of the PFECA and PFESA subgroups, namely PFMOAA, PFMOPrA, PFMOBA, PFO2HpA, PFO2DA, PFO3DA, PFO3TriDA, PFoxaOA, branched ADONA, PF2EOESA, 8:2 Cl-PFESA, Nafion-BP1 and similar to Nafion-BP2. Thus, our study substantially expands the mechanistic transcriptomic evidence for ether-PFAS and shows that these compounds, despite structural differences from legacy PFCA/PFSA congeners, largely induce the same core hepatic signatures. Another important aspect of our dataset is the inclusion of short-chain and very short-chain PFAS, which are still underrepresented in mechanistic studies. In our DGE analysis, some of these compounds – most notably PFPrA and PFMOAA – induced only minor transcriptional changes even at the “high” concentration category, and this was mirrored at the pathway level, where compounds with weak overall transcriptomic responses (PFUnA, PFOS and similar to Nafion-BP2) also showed only minor IPA pathway perturbations. In particular, the additional pathway groups beyond the shared core response were not broadly altered across all PFAS, but mainly by stronger responders at the “high” concentration; this included several immune-related pathways as well as pathways related to translation/transcription, amino acid metabolism, and cell division. Since many of these pathway classes were absent at the corresponding “medium” and “low” concentrations, they are best interpreted as a secondary response layer that emerges mainly under stronger, near-cytotoxic perturbation conditions. Therefore, the absence of these broader pathway changes for short-chain PFAS should not be interpreted as biological inactivity, but rather as lower apparent potency and/or lower effective intracellular exposure within the tested concentration window (maximum 250 µM), such that mainly the shared core response is captured while broader secondary responses remain below the threshold of detection. This interpretation is also relevant for PFOS and PFUnA, which showed only limited transcriptional responsiveness in our dataset despite known bioactivity in other systems. Although this remains a working hypothesis that should be tested in subsequent studies, a plausible explanation is that these compounds may have had a reduced freely dissolved (bioavailable) fraction in the in vitro assay because of strong interfacial adsorption and partitioning behavior (consistent with high surface activity), thereby lowering the effective intracellular concentration.

At the same time, according to IPA, the most robust alterations in gene expression induced by most of the 33 PFAS were the upregulation of PPARα-dependent target genes and in turn PPARα-mediated regulation of fatty acid and lipid metabolism. This was observed for 29 of the 33 tested PFAS, and on a broader concentration range. PFAS-mediated activation of PPARα has been shown at several levels and for several species, e.g., by transactivation assays (Behr et al. 2020b; Wolf et al. 2008, 2012), PPARα-binding assays (Ishibashi et al. 2019), by molecular docking studies (Khazaee et al. 2021; Kowalska et al. 2023), etc. These studies were usually restricted to PFCA and PFSA congeners, however, the recent transcriptomic studies mentioned above as well as our own study indicate that activation of PPARα seems to be a common feature shared by most PFAS of various PFAS subgroups. We have recently shown by means of a transactivation assay that all PFAS of our test set except for PFDA, PFUnA and 8:2 Cl-PFESA were capable of activating PPARα (Alker et al. 2026). In that study, PFECA congeners were shown to be the most potent PPARα agonists, and PFAS with a sulfonic acid functional group were less potent than PFAS with a carboxylic acid group. This is in line with previous reports showing that PFSA congeners are less potent PPARα agonists compared to PFCA congeners (Behr et al. 2020b; Rosenmai et al. 2018; Wolf et al. 2008, 2012). Thus, within the AOP framework, PFAS-mediated PPARα activation can be considered an important molecular initiating event in hepatocytes, and disturbance of PPARα-regulated fatty acid and lipid metabolism is likely a major mechanism underlying PFAS-induced hepatocellular effects.

In addition to PPARα activation, most of the 33 PFAS congeners affected xenobiotic metabolism in HepaRG cells, mainly regulated via PXR and/or CAR. In line with our own results, gene expression related to xenobiotic metabolism regulated by CAR was also affected by most PFAS in the Addicks study (Addicks et al. 2025). Moreover, activation of PXR and/or CAR by a number of legacy and novel PFAS has been reported for several mouse studies as summarized in Robarts et al. (2024). In the present study, expression of many genes of the CYP, GST and UGT families was strongly affected by most PFAS, however, the direction of regulation was not as clear as for the upregulation of the PPARα target genes. Thus, PFAS-induced modulation of phase I and phase II gene expression is putatively not exclusively regulated by PXR and CAR, and additional factors may account for the impact of PFAS on xenobiotic metabolism in HepaRG cells. This is mechanistically plausible, particularly for CAR, because CAR-dependent xenobiotic responses can also arise through indirect activation mechanisms involving signaling-dependent control of CAR phosphorylation status (e.g., Thr38 dephosphorylation) and nuclear translocation (Mutoh et al. 2013), rather than direct receptor-ligand interaction. In addition, both CAR and PXR act as heterodimers with RXR, and PPARα is also an RXR partner, making RXR-centered nuclear receptor crosstalk a plausible contributor to the mixed metabolic/xenobiotic signature observed here. Indeed, we have previously shown that a number of PFCA and PFSA congeners neither activate PXR nor CAR in a transactivation assay whereas they clearly activated PPARα (Behr et al. 2020b), pointing to a molecular mechanism of xenobiotic metabolism activation that is possibly independent of PXR and/or CAR. Although the exact molecular initiating event remains unclear, the cellular detoxification programs seem to be activated upon exposure to various PFAS congeners. However, these programs are not effective in the case of PFAS as these compounds are resistant to metabolic conversion, and they are probably no substrates of phase I or phase II enzymes. Thus, HepaRG cells seem to be capable to recognize PFAS as xenobiotics, but they fail to convert them via phase I and phase II enzymes to facilitate cellular excretion.

In addition to its implications for xenobiotic detoxification, this RXR-/PXR-/CAR-linked response is likely relevant for the broader metabolic phenotype observed here, because these nuclear receptor networks are closely connected to hepatic lipid, cholesterol and bile acid homeostasis and may therefore contribute to the pronounced PFAS-associated effects on *CYP7A1* expression and bile acid/cholesterol biosynthesis discussed below. In agreement with our data, inhibition of cholesterol biosynthesis by a number of legacy PFAS was a key finding of several other transcriptomic studies conducted with HepaRG cells (Louisse et al. 2020, 2023) and primary human hepatocytes (Addicks et al. 2025). In HepaRG cells, however, it has been shown before that cholesterol levels were not affected by PFOA and PFOS (Behr et al. 2020a; Louisse et al. 2020). On the other hand, it has been frequently reported that legacy PFAS induce a strong downregulation of *CY7A1* gene expression in primary human hepatocyte spheroids and in HepaRG cells (Fig. 6; Addicks et al. 2025; Behr et al. 2020a; Louisse et al. 2023) that, at least in HepaRG cells, leads to a strong decrease of CYP7A1 protein (Behr et al. 2020a). CYP7A1 catalyses the first, rate-limiting step to produce bile acids from cholesterol in hepatocytes, and we have previously shown that, indeed, levels of certain bile acids are strongly decreased in HepaRG cell cultures upon PFOA or PFOS treatment (Behr et al. 2020a). Thus, the observed inhibition of gene expression of cholesterol biosynthesis genes is probably feedback of PFAS-induced *CYP7A1* downregulation to maintain cellular cholesterol levels. In their study, Addicks et al. suggested that integration of the long-chain PFAS into cellular membranes may lead to inhibition of the SCAP/SREBP regulatory system and in turn to the downregulation of cholesterol biosynthesis genes (Addicks et al. 2025). We favor the hypothesis that in human hepatocytes, the conversion of cholesterol to bile acids is strongly inhibited upon PFAS-induced *CYP7A1* downregulation, resulting in a decreased cholesterol biosynthesis of the cells to avoid production of excess cholesterol. Thus, the repeated observation that PFAS inhibit cholesterol biosynthesis at the transcriptomic level in human hepatocytes may in fact be a result of PFAS-mediated inhibition of bile acid synthesis via *CYP7A1* downregulation. In the present study, an association between PFAS levels and bile acid biosynthesis is also reflected by the IPA prediction of intrahepatic cholestasis being the most significant downstream effect induced by PFAS (Fig. 4). Cholestasis is a liver disease that is based on an imbalance of hepatic bile acid synthesis and secretion. Although a correlation between PFAS levels and cholestatic diseases has not been shown so far for humans, epidemiological studies have at least revealed a positive association between PFAS blood serum levels and serum cholesterol (Dong et al. 2019; Fu et al. 2014; Jain and Ducatman 2019; Nelson et al. 2010). At first glance, this finding seems to contradict the observations at the molecular and cellular levels of a PFAS-mediated inhibition of cholesterol biosynthesis in human hepatocytes. Again, a feedback mechanism may be assumed that is active in liver to repress biosynthesis of excess cholesterol in response to PFAS-induced increase of cholesterol levels, probably due to PFAS-mediated inhibition of CYP7A1-regulated bile acid synthesis. Interestingly, it is the exact opposite in rodents. Numerous animal studies have shown that PFAS application results in decreased serum cholesterol levels in mouse (Minata et al. 2010; Wang et al. 2014) and rat (NTP 2019a, 2019b). At the molecular level, however, cholesterol biosynthesis was shown to be activated in mouse liver at the gene expression level by a number of PFAS as summarized by Robarts et al. (2024). Taken together, PFAS seem to impact bile acid biosynthesis in connection with cholesterol homeostasis, however, the underlying molecular mechanisms as well as the interspecies differences are not yet fully understood.

In our study, a number of additional canonical pathways associated with, e.g., amino acid metabolism, cell division, general transcription and translation, and immune response, were affected by some PFAS, however, these effects were only observed at the “high” concentration (Fig. 2) that was close to the cytotoxicity threshold of these PFAS congeners. Thus, these findings may be interpreted as massive disturbances in cellular signaling and metabolism that seem to occur at concentrations close to those that induce cell death. Addicks et al. (2025) reported an impact of certain PFAS on gene expression related to, e.g., immune functions, protein synthesis (ribosome and initiation), and citric acid cycle in human liver spheroids. These effects were observed for long-chain PFCA and PFSA congeners. In the Addicks study, 100 µM was the highest test concentration, which may be close to the cytotoxicity threshold of these PFAS congeners. It may be assumed that the shorter-chain PFAS would possibly also affect these additional pathways, if tested at concentrations higher than 100 µM and close to the respective cytotoxicity threshold. In the present study, 250 µM was selected as the highest test concentration although the cytotoxicity threshold was much higher for many PFAS, in particular for the short-chain PFCA and PFSA congeners. These PFAS did not induce alterations in gene expression associated with, e.g., immune or ribosome functions, which is in line with the results of the Addicks study. In contrast to the Addicks study, however, we suggest that this is not due to the lower membrane integration potential of these shorter-chain PFAS, but that this is simply due to the fact that the cytotoxicity threshold is not yet reached for these PFAS at these concentrations. Finally, it has to be noted that the short-chain PFAS have much lower bioaccumulative potential than the long-chain congeners. Whereas short-chain congeners are completely cleared by kidney, long-chain congeners such as PFOA and PFOS are partly cleared by liver and excreted with bile (Abraham et al. 2024). Thus, future studies may focus on kidney toxicity of short-chain PFAS including in vitro studies with renal cell lines.

Regarding the test concentrations, it has to be noted that relatively high PFAS concentrations were used in the present in vitro study (range 5–250 µM) which is three to four orders of magnitude above the PFAS blood serum levels in the general population. As an example, PFOS blood serum levels of the general population have frequently been reported to be in a range of about 10 nM, and it is well-accepted that PFOS induces adverse health effects in humans at the sub-micromolar level (EFSA 2020). Many PFAS are known to display steep dose-response curves with respect to numerous in vivo and in vitro toxicological endpoints. This also accounts for the present study in which each of the 33 PFAS congeners was tested at three non-cytotoxic concentrations, and in which hardly any response at the lowest test concentrations was observed (Fig. 1). Thus, the use of high PFAS test concentrations is necessary in in vitro studies to obtain insights into the PFAS-driven molecular toxicity mechanisms which supports hazard characterization of these compounds, but interpretation of the in vitro results with respect to real-life exposure scenarios should be handled with care. However, in the present study, the differentiated HepaRG cells even did not strongly respond to incubation with 100 µM PFOS, which was the highest, non-cytotoxic concentration in our hands. Due to cytotoxicity issues, we have also used maximal 100 µM PFOS for 24 h HepaRG treatments (Sadrabadi et al. 2024) and maximal 50 µM for 48 h treatments (Behr et al. 2020a) in our previous studies. Louisse et al. used up to 400 µM PFOS for HepaRG treatments in their studies (Louisse et al. 2020, 2023), and they observed massive alterations in gene expression at 200 µM and 400 µM PFOS. At 100 µM, however, PFOS had minor effects on gene expression in HepaRG cells also in the Louisse study (Louisse et al. 2023). Based on their cytotoxicity data, Addicks et al. used maximal 20 µM PFOS for the 10-days exposure of human liver spheroids and observed a strong impact of PFOS on gene expression (Addicks et al. 2025). This illustrates that the impact of PFOS on, e.g., gene expression depends on the cell model, the exposure duration, the incubation concentration, and probably additional technical details of the different cell culture protocols. Moreover, the use of different cut-off criteria in the evaluation of the transcriptomic data accounts for the seemingly varying potency of PFOS in the different studies. In addition to PFOS, this may also apply to the apparently different impact of PFNA, PFDA and PFUnA on gene expression in the different studies.

In conclusion, the different transcriptomic studies consistently revealed that a broad range of different PFAS congeners share comparable molecular mechanisms of action in human hepatocytes. At the gene expression level, PFAS commonly induce fatty acid and lipid metabolism via PPARα activation, and affect xenobiotic metabolism, at least in part through PXR/CAR-associated signaling. In the present study, this was shown for a total of 33 PFAS congeners, including 13 congeners of the PFECA/PFESA subgroups, for which such transcriptomic evidence in HepaRG is reported here for the first time. Importantly, the present dataset also included short-chain and very short-chain PFAS, which are still underrepresented in mechanistic studies. These compounds generally induced weaker transcriptomic responses and fewer additional pathway perturbations, but this should not be interpreted as biological inactivity. Rather, our data support the view that short-chain PFAS can contribute to the same core hepatocellular transcriptomic signature, whereas broader secondary responses may remain below detection within the tested concentration window. Additional canonical pathways and related upstream regulators were affected by a subset of PFAS, but mainly at high, near-cytotoxic concentrations, linking these findings to broader metabolic and regulatory disturbances that are likely not specific to PFAS. Interestingly, cholesterol and bile acid biosynthesis were strongly inhibited by several PFAS, probably due to pronounced PFAS-mediated downregulation of *CYP7A1* gene expression. Although these effects were observed only at high micromolar PFAS concentrations in our HepaRG in vitro system, they nevertheless provide mechanistic insight that may explain the observed associations between PFAS serum levels and serum cholesterol level in epidemiological studies. Overall, these findings highlight the value of transcriptomic studies for resolving both shared and compound-dependent features of PFAS hepatotoxicity and for generating testable mechanistic hypotheses at the molecular level.

## Supplementary Information

Below is the link to the electronic supplementary material.

Supplementary Material 1

Supplementary Material 2

## Data Availability

Transcriptomics raw data are available from the European Nucleotide Archive (ENA) under accession number PRJEB104003.
