# Supplementary material for: Analysis of transcriptomic alterations induced by 33 different per- and polyfluoroalkyl substances (PFAS) in differentiated HepaRG cells
Source: Arch Toxicol. 2026 May 13;100(9):4021–39. doi: 10.1007/s00204-026-04429-5 (PMC13454013; doi:10.1007/s00204-026-04429-5)
Supplement: Supplementary file 1 [file 204_2026_4429_MOESM1_ESM.pdf]

Analysis of transcriptomic alterations induced by 33 different per- and polyfluoroalkyl substances (PFAS) in differentiated HepaRG cells

Heike Sprenger[1], Wiebke Alker[1], Anna Rocchi[2], Chiara Leo[2], Rosa Giglio[2], Greta Immobile Molaro[2], Francesco Dondero[3], Albert Braeuning[1], Thorsten Buhrke[1#]

[1] German Federal Institute for Risk Assessment (BfR), Department Chemical and Product Safety, Max-Dohrn-Str. 8-10, 10589 Berlin, Germany

[2] NGS and Bioinformatic Laboratory – Polo d'Innovazione di Genomica, Genetica e Biologia SRL, Strada del Petriccio e Belriguardo 35-53100, Siena, Italy.

[3] Department of Science and Technological Innovation, University of Eastern Piedmont Alessandria Novara Vercelli, Viale Michel 11, 15121 Alessandria, Italy.

[#] Corresponding Author:

Dr. Thorsten Buhrke, German Federal Institute for Risk Assessment (BfR), Department Chemical and Product Safety, Max-Dohrn-Str. 8-10, 10589 Berlin, Germany; E-Mail thorsten.buhrke@bfr.bund.de, phone 0049 30 18412-77401

Supplementary Figures 1 – 17

Supplementary Figure 1. PFAS cytotoxicity in differentiated HepaRG cells.

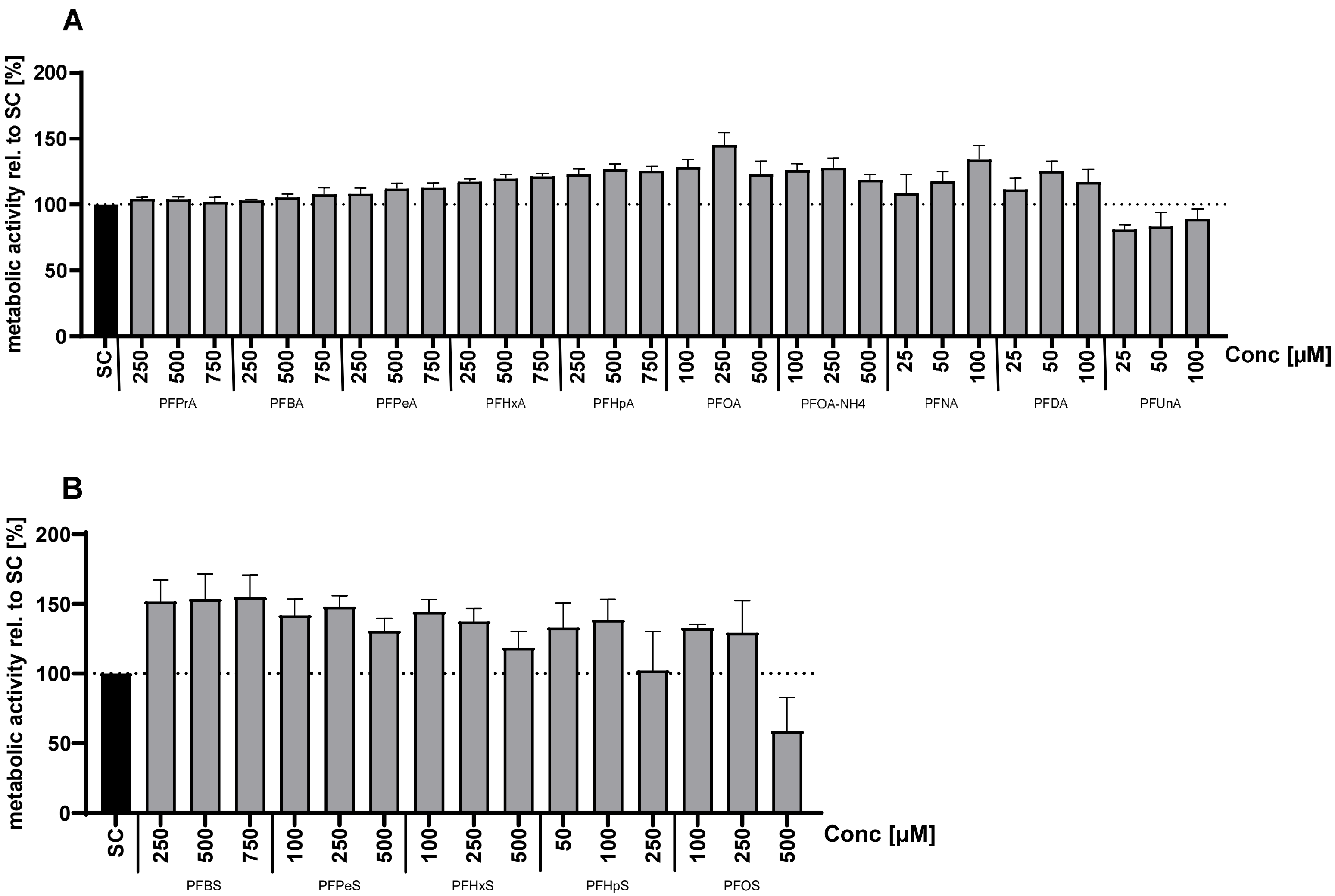

C

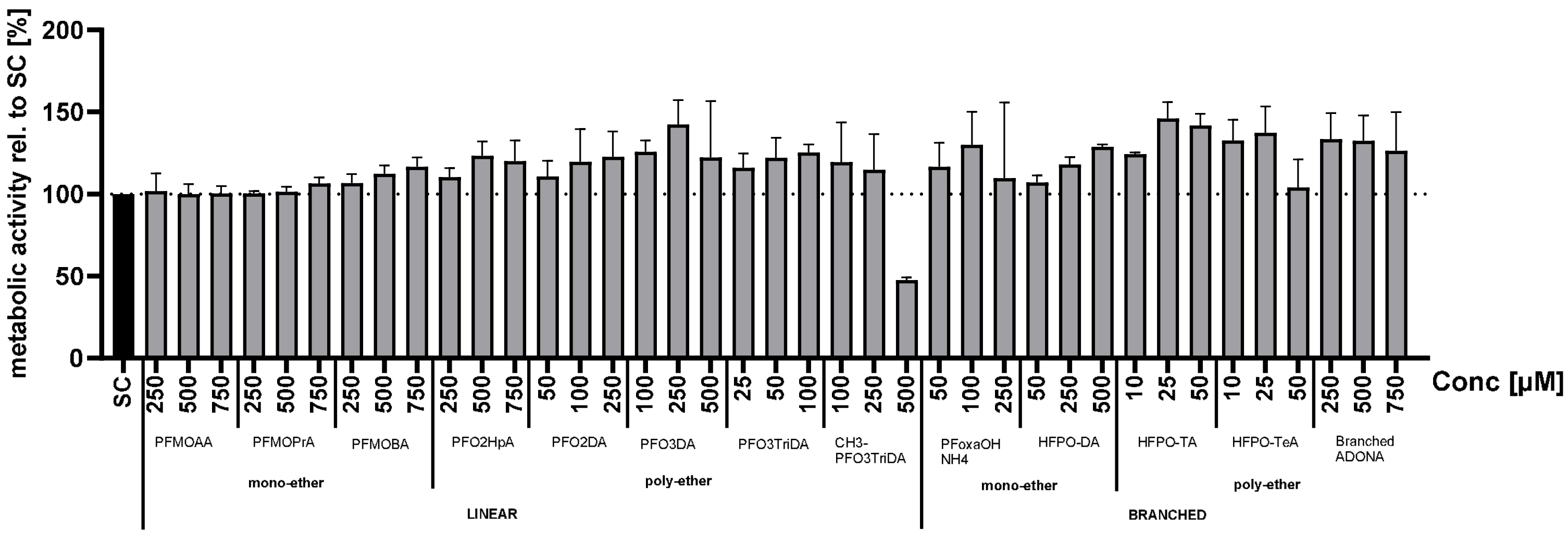

D

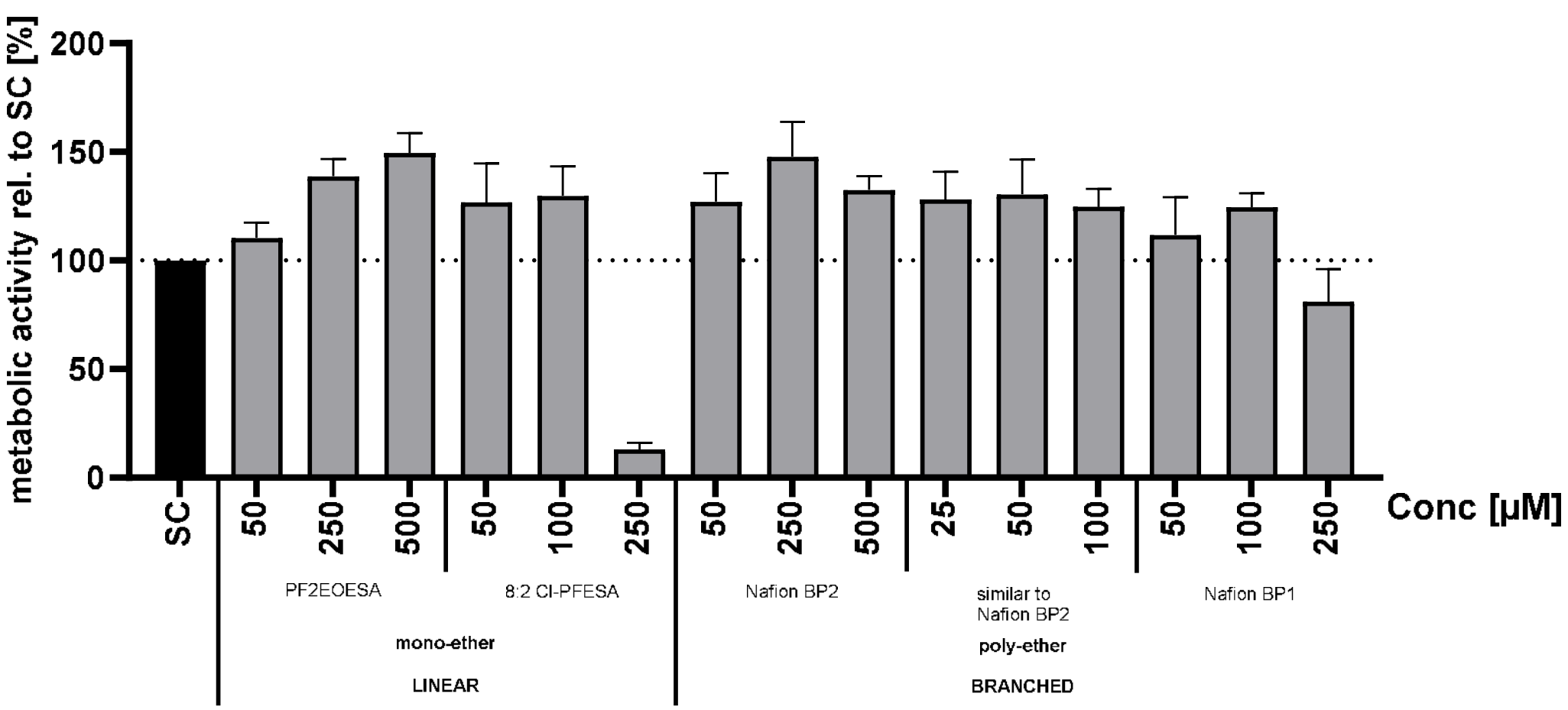

Supplementary Figure 1. Cellular viability relative to untreated solvent control of HepaRG cells, determined by MTT-assay after 24 h of incubation with PFAS congeners (A: PFCA, B: PFSA; C: PFECA, D: PFESA) and concentrations as indicated. Data are shown as mean + standard deviation of three biological replicates with each three technical replicates.

Supplementary Figure 2. IPA canonical pathways, “medium” concentration category

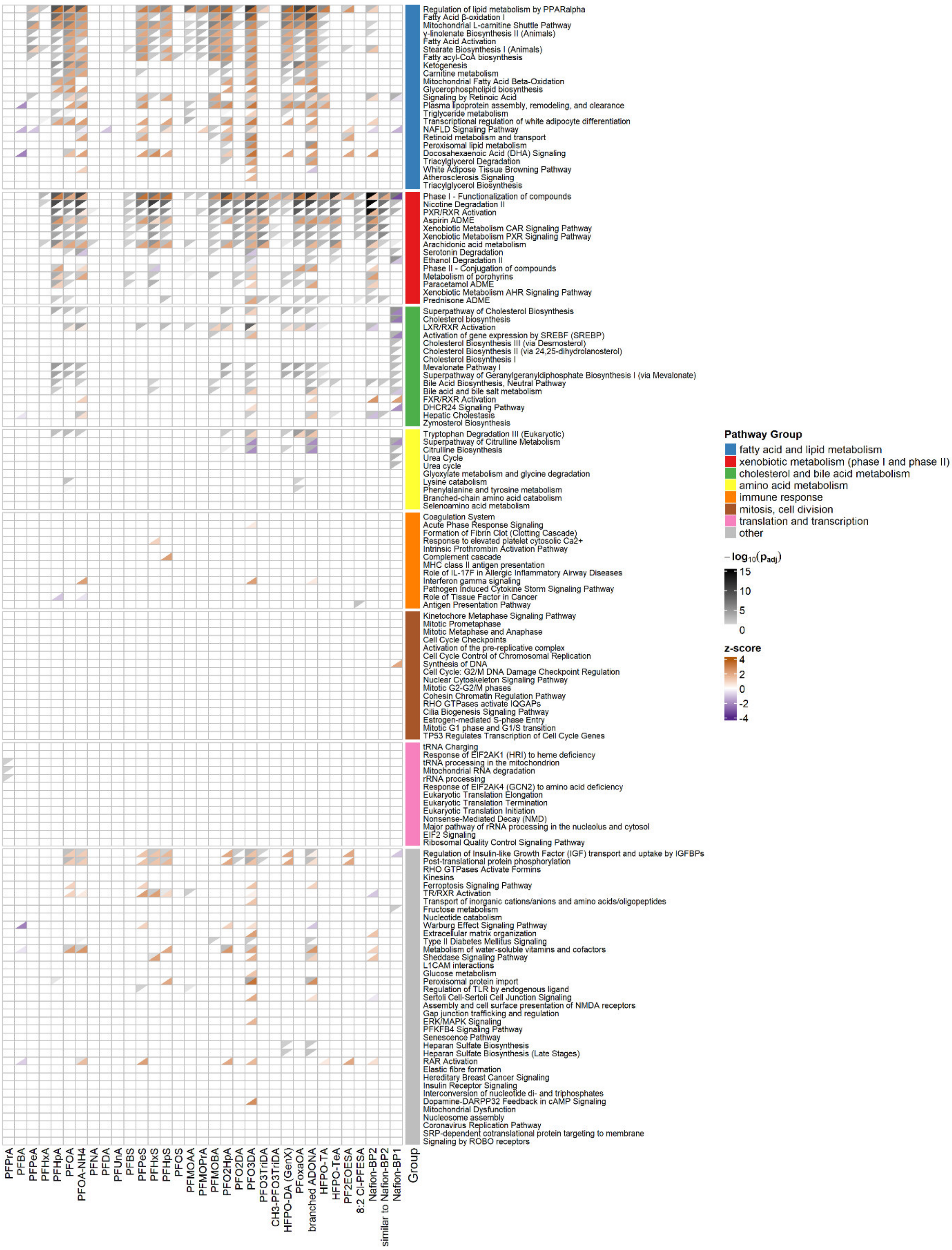

Supplementary Figure 2. Comparison analysis of the IPA canonical pathways based on the data from the “medium” concentration of the 33 PFAS. The pathways are presented in the same order as for the “high” concentration category (Fig. 2). When $p_{adj} < 0.05$, it is presented in light-gray to black with increasing $-\log_{10}(p_{adj})$-values at the upper left triangles of the heatmap tiles. At the lower right triangles of the heatmap tiles, the IPA z-scores are given in orange (meaning activation of the respective pathway) and violet (meaning inhibition of the respective pathway).

Supplementary Figure 3. IPA canonical pathways, "low" concentration category

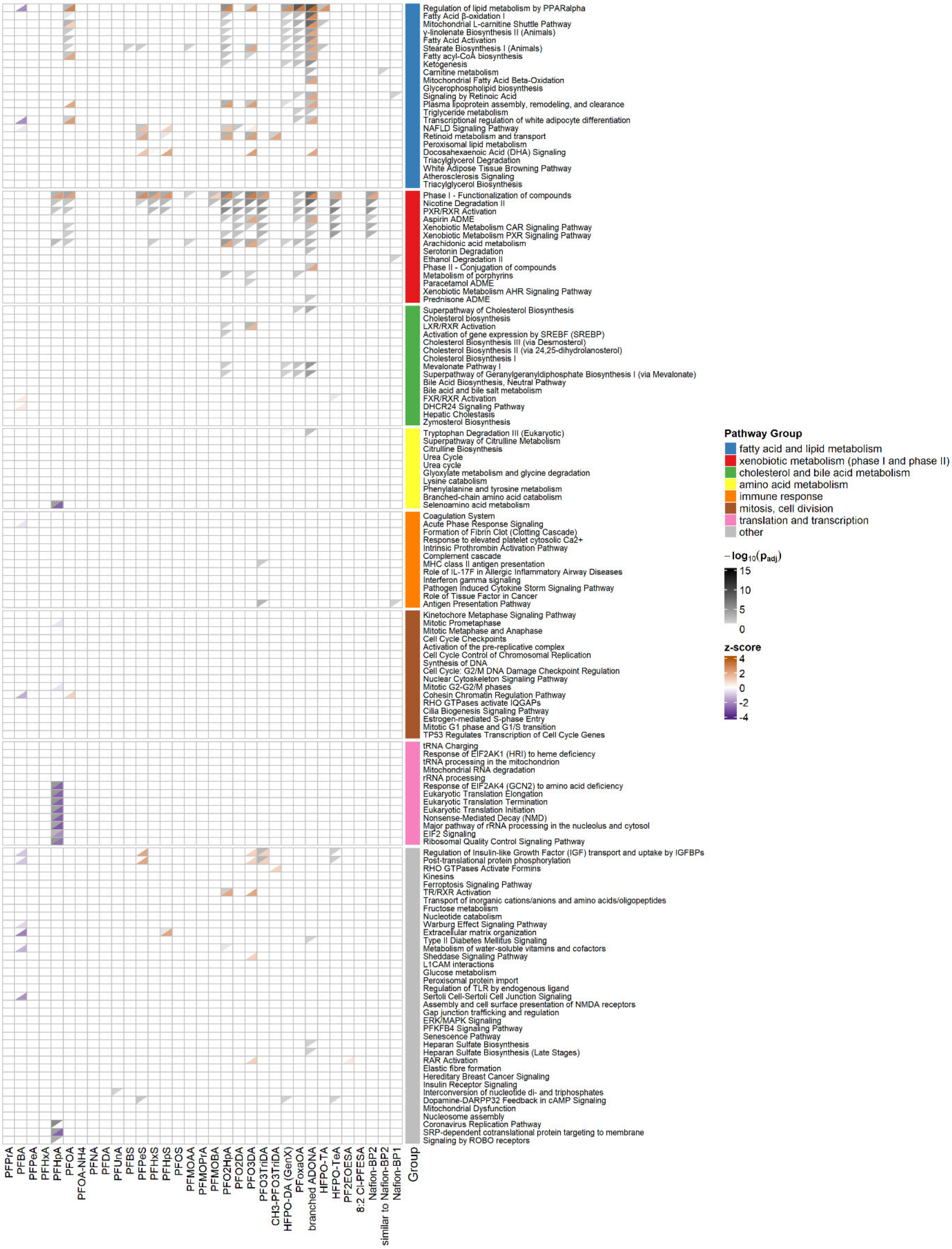

Supplementary Figure 3. Comparison analysis of the IPA canonical pathways based on the data from the "low" concentration of the 33 PFAS. The pathways are presented in the same order as for the "high" concentration category (Fig. 2). When $p_{adj} < 0.05$, it is presented in light-gray to black with increasing $-\log_{10}(p_{adj})$-values at the upper left triangles of the heatmap tiles. At the lower right triangles of the heatmap tiles, the IPA z-scores are given in orange (meaning activation of the respective pathway) and violet (meaning inhibition of the respective pathway).

Supplementary Figure 4. IPA upstream regulators, "medium" concentration category

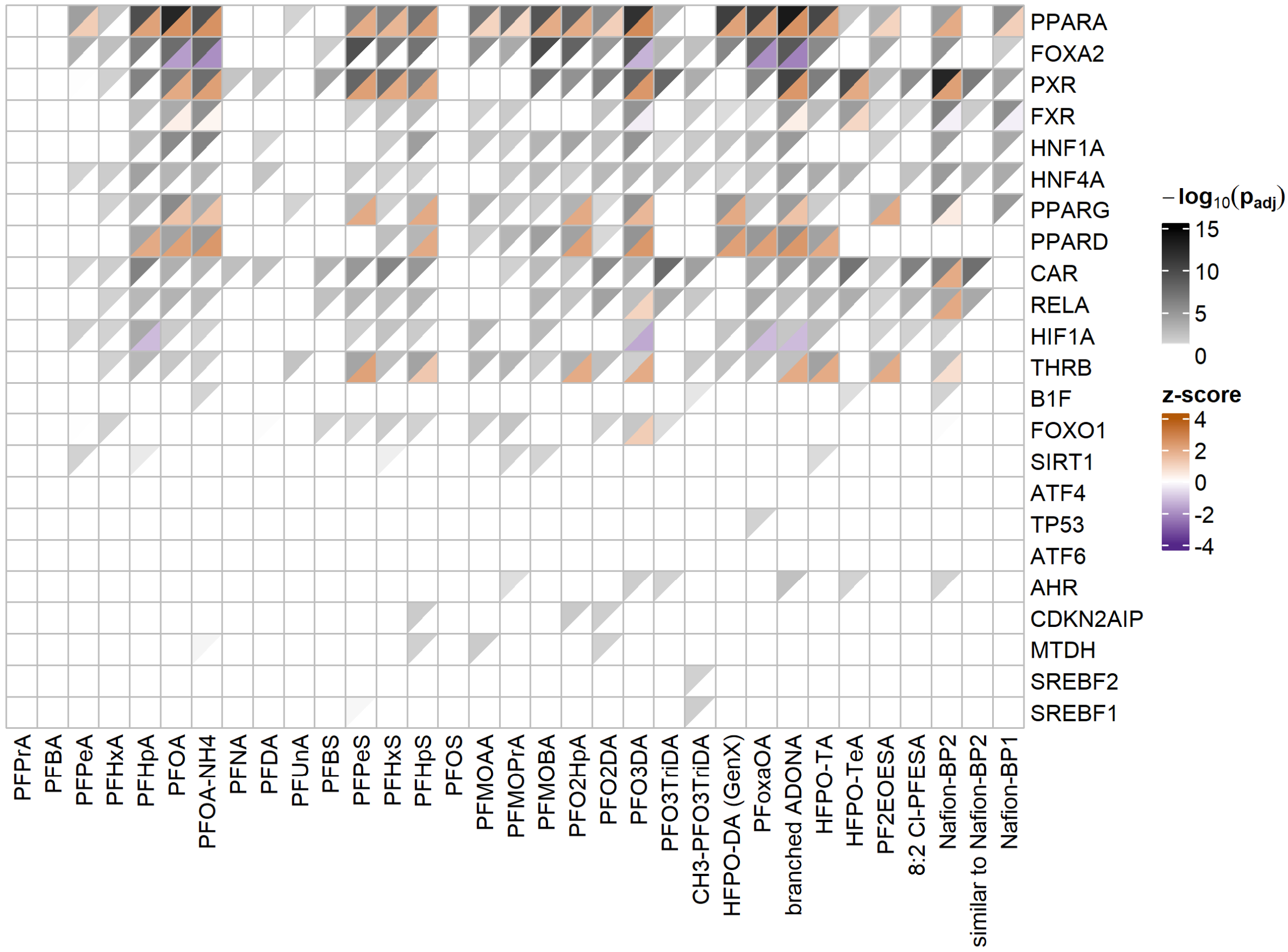

Supplementary Figure 4. Comparison analysis of the IPA upstream regulators based on the data from the "medium" concentration of the 33 PFAS. The upstream regulators are presented in the same order as for the "high" concentration category (Fig. 3). When $p_{adj} < 0.05$, it is presented in light-gray to black with increasing $-\log_{10}(p_{adj})$-values at the upper left triangles of the heatmap tiles. At the lower right triangles of the heatmap tiles, the IPA z-scores are given in orange (meaning activation of the respective upstream regulator) and violet (meaning inhibition of the respective upstream regulator).

Supplementary Figure 5. IPA upstream regulators, "low" concentration category

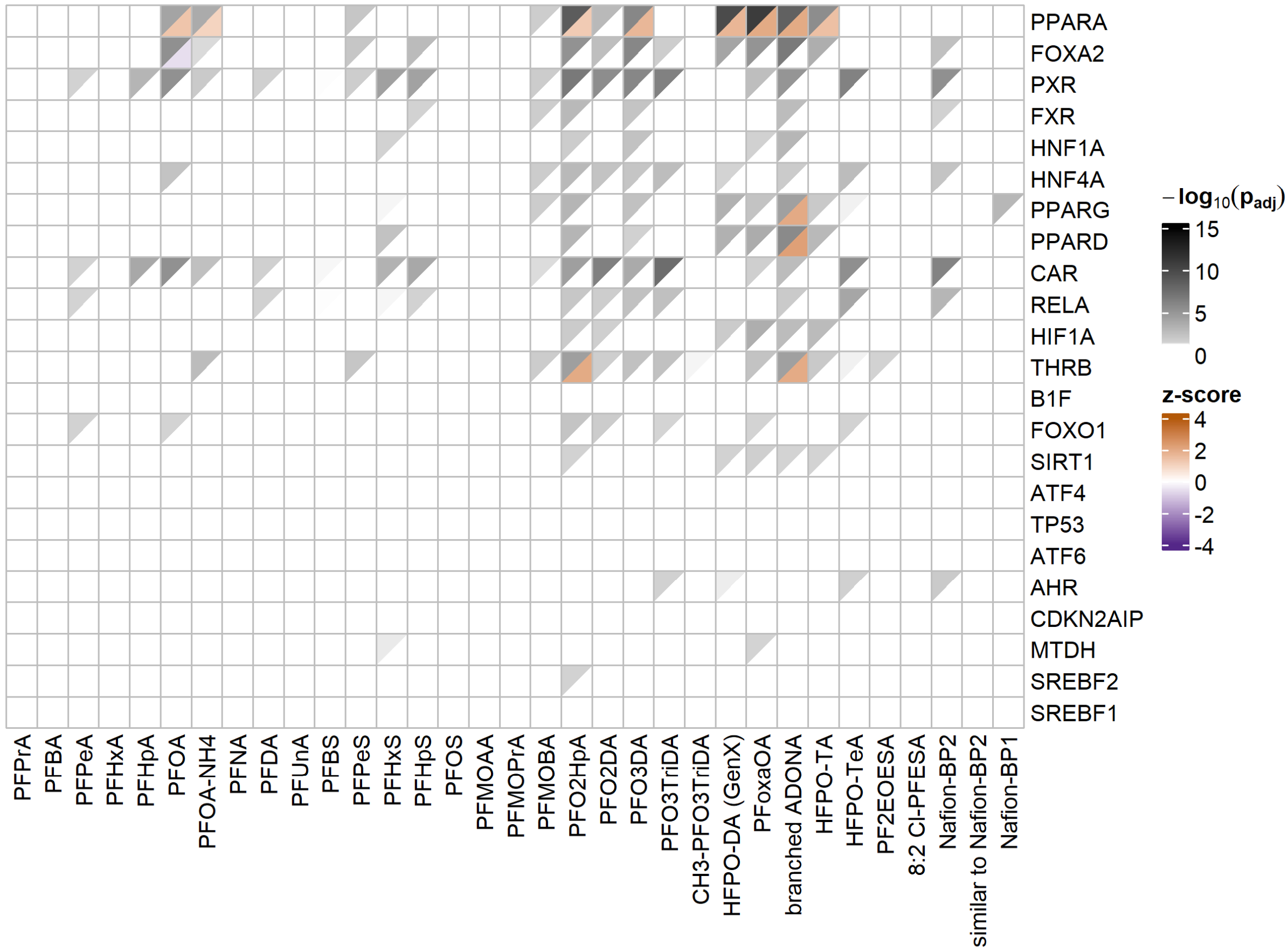

Supplementary Figure 5. Comparison analysis of the IPA upstream regulators based on the data from the "low" concentration of the 33 PFAS. The upstream regulators are presented in the same order as for the "high" concentration category (Fig. 3). When $p_{adj} < 0.05$, it is presented in light-gray to black with increasing $-\log_{10}(p_{adj})$-values at the upper left triangles of the heatmap tiles. At the lower right triangles of the heatmap tiles, the IPA z-scores are given in orange (meaning activation of the respective upstream regulator) and violet (meaning inhibition of the respective upstream regulator).

Supplementary Figure 6. IPA downstream effects (tox functions), “medium” concentration category

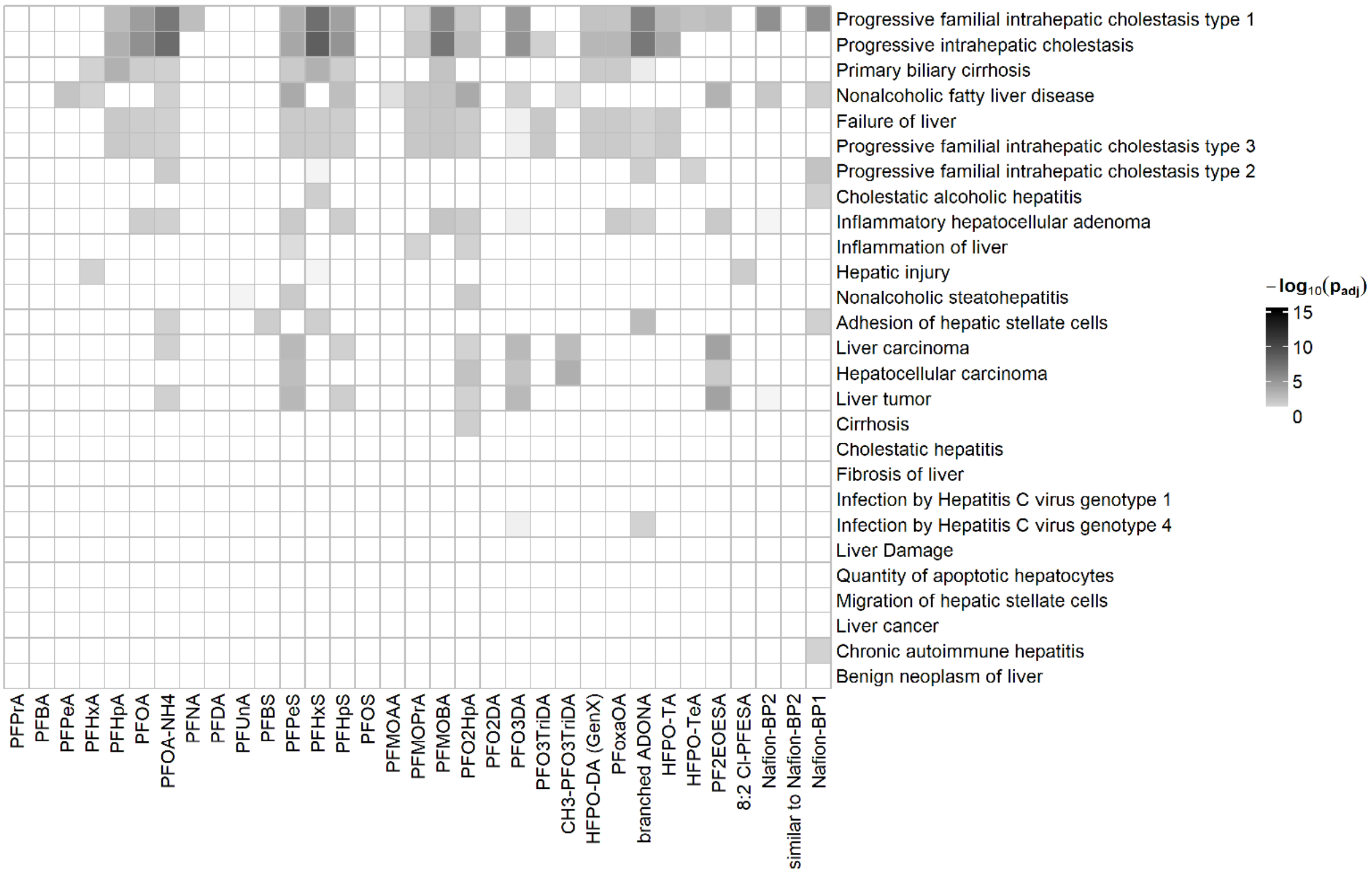

Supplementary Figure 6. Comparison analysis of the IPA downstream effects (tox functions) based on the data from the “medium” concentration of the 33 PFAS. The downstream effects are presented in the same order as for the “high” concentration category (Fig. 4). When $p_{adj} < 0.05$, it is presented in light-gray to black with increasing $-\log_{10}(p_{adj})$-values.

Supplementary Figure 7. IPA downstream effects (tox functions), “low” concentration category

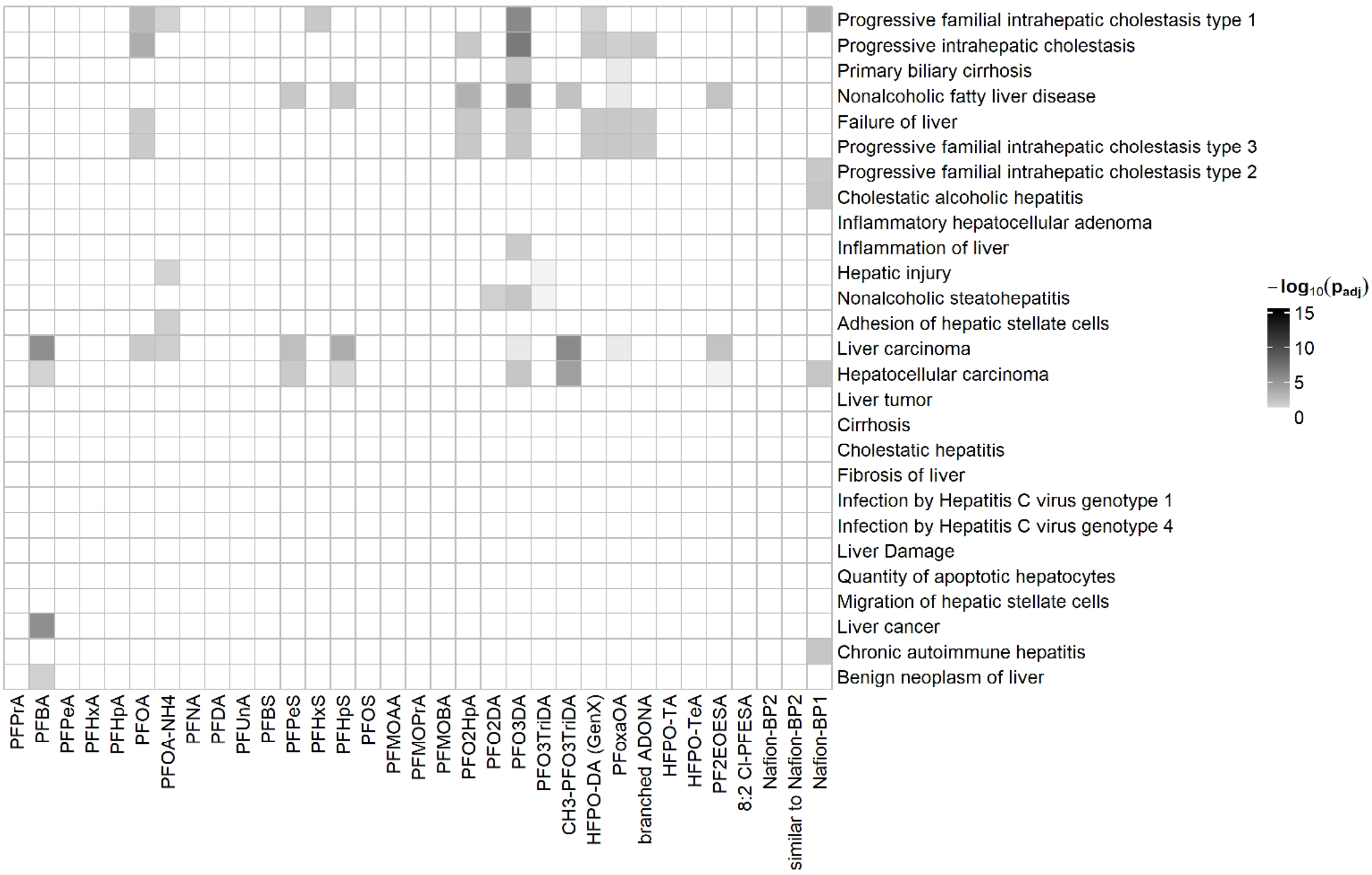

Supplementary Figure 7. Comparison analysis of the IPA downstream effects (tox functions) based on the data from the “low” concentration of the 33 PFAS. The downstream effects are presented in the same order as for the “high” concentration category (Fig. 4). When $p_{adj} < 0.05$, it is presented in light-gray to black with increasing $-\log_{10}(p_{adj})$-values.

Supplementary Figure 8.

Genes of the IPA canonical pathway “Regulation of lipid metabolism by PPARalpha”, “high” concentration category

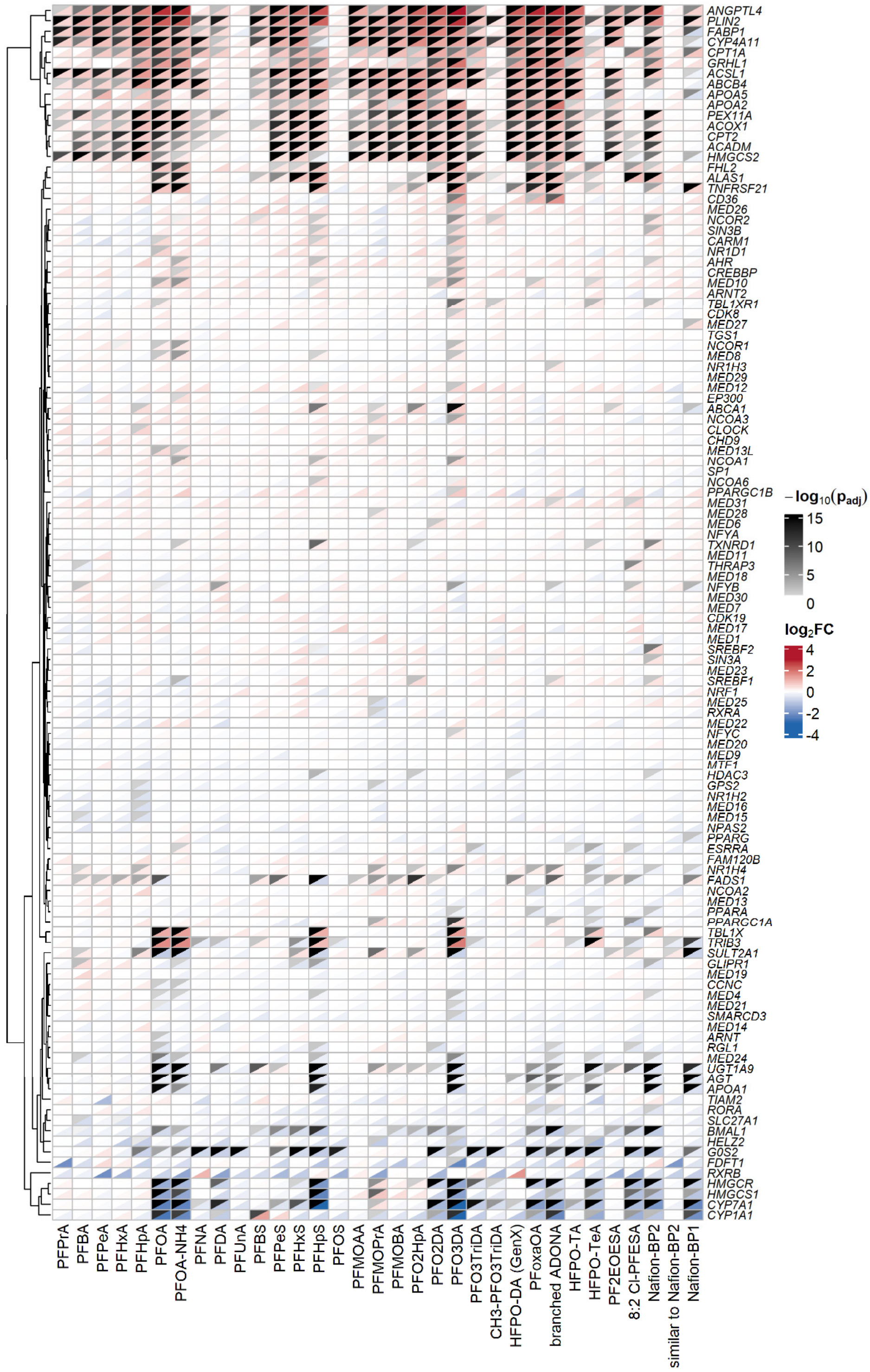

Supplementary Figure 8. Comparison analysis of the genes of the IPA canonical pathway “Regulation of lipid metabolism by PPARalpha” based on the data from the “high” concentration of the 33 PFAS. All genes that belong to the IPA-pathway “Regulation of lipid metabolism by PPARalpha” are displayed. The genes were sorted according to $\log_2FC$ from positive values at the top to negative values at the bottom. When $p_{adj} < 0.05$, it is presented in light-gray to black with increasing $-\log_{10}(p_{adj})$-values at the upper left triangles of the heatmap tiles. At the lower right triangles of the heatmap tiles, $\log_2FC$ are given in red (meaning upregulation of expression of the respective gene) and blue (meaning downregulation of expression of the respective gene).

Supplementary Figure 9.

Genes of the IPA canonical pathway “Regulation of lipid metabolism by PPARalpha”, “medium” concentration category

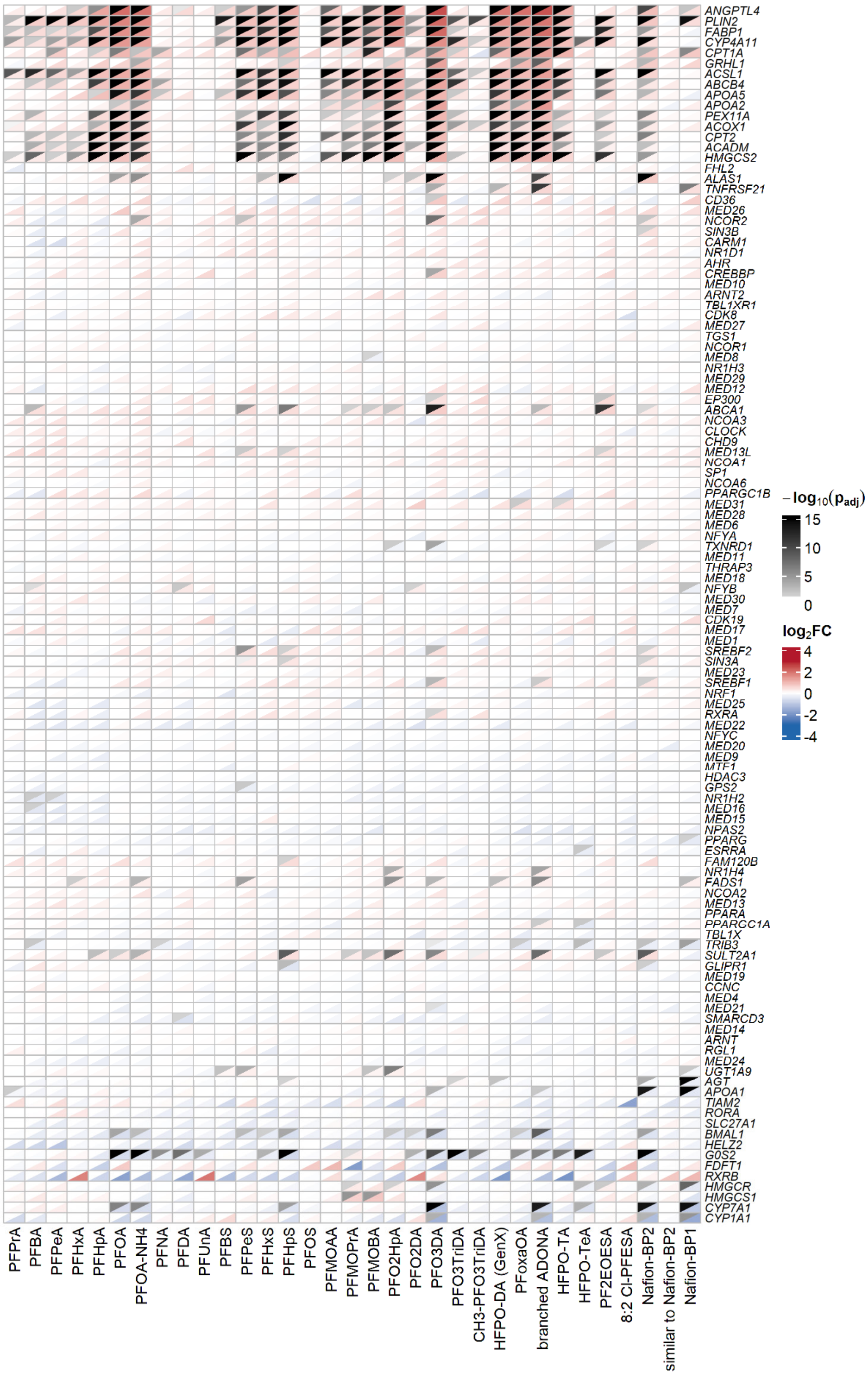

Supplementary Figure 9. Comparison analysis of the genes of the IPA canonical pathway “Regulation of lipid metabolism by PPARalpha” based on the data from the “medium” concentration of the 33 PFAS. All genes that belong to the IPA-pathway “Regulation of lipid metabolism by PPARalpha” are displayed. The genes are presented in the same order as for the “high” concentration category (Supplementary Figure 8). When $p_{adj} < 0.05$, it is presented in light-gray to black with increasing -$\log_{10}(p_{adj})$-values at the upper left triangles of the heatmap tiles. At the lower right triangles of the heatmap tiles, $\log_2FC$ are given in red (meaning upregulation of expression of the respective gene) and blue (meaning downregulation of expression of the respective gene).

Supplementary Figure 10.

Genes of the IPA canonical pathway "Regulation of lipid metabolism by PPARalpha", "low" concentration category

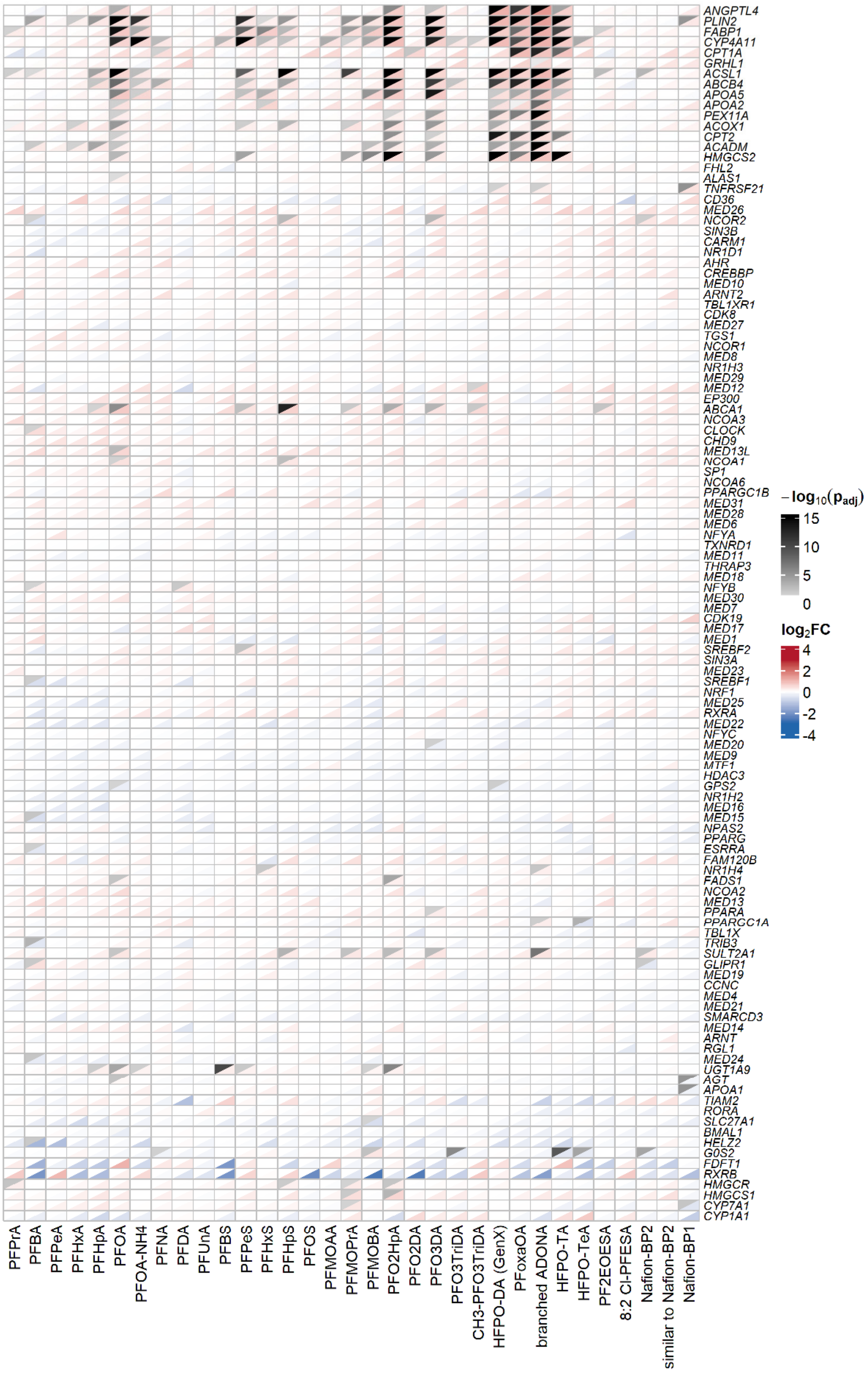

Supplementary Figure 10. Comparison analysis of the genes of the IPA canonical pathway "Regulation of lipid metabolism by PPARalpha" based on the data from the "low" concentration of the 33 PFAS. All genes that belong to the IPA-pathway "Regulation of lipid metabolism by PPARalpha" are displayed. The genes are presented in the same order as for the "high" concentration category (Supplementary Figure 8). When $p_{adj} < 0.05$, it is presented in light-gray to black with increasing -$\log_{10}(p_{adj})$-values at the upper left triangles of the heatmap tiles. At the lower right triangles of the heatmap tiles, $\log_2FC$ are given in red (meaning upregulation of expression of the respective gene) and blue (meaning downregulation of expression of the respective gene).

Supplementary Figure 11.

Genes of the IPA canonical pathway "phase I – functionalization of compounds", "high" concentration category

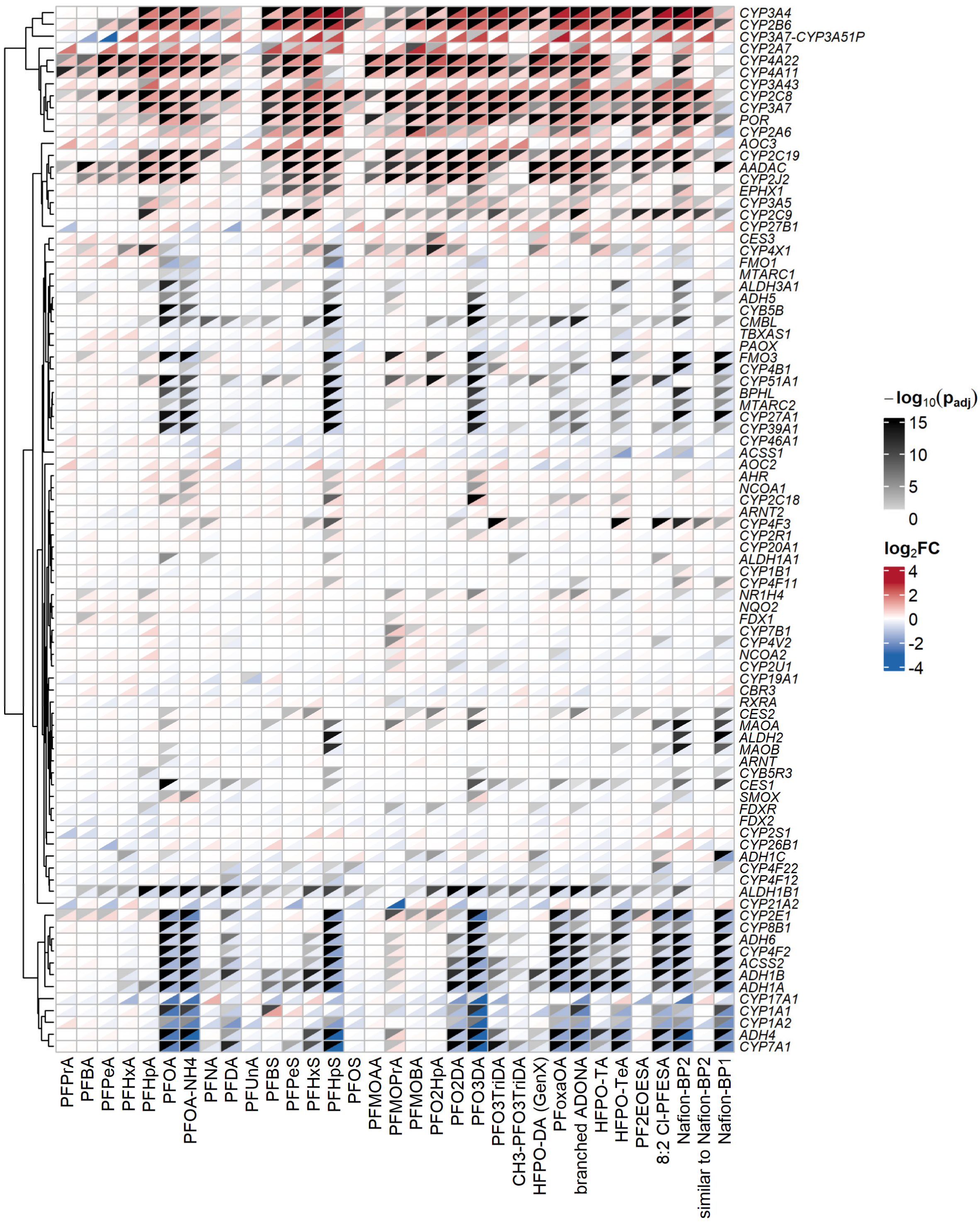

Supplementary Figure 11. Comparison analysis of the genes of the IPA canonical pathway "phase I – functionalization of compounds" based on the data from the "high" concentration of the 33 PFAS. All genes that belong to the IPA-pathway "phase I – functionalization of compounds" are displayed. The genes were sorted according to $\log_2$FC from positive values at the top to negative values at the bottom. When $p_{adj} < 0.05$, it is presented in light-gray to black with increasing -$\log_{10}(p_{adj})$-values at the upper left triangles of the heatmap tiles. At the lower right triangles of the heatmap tiles, $\log_2$FC are given in red (meaning upregulation of expression of the respective gene) and blue (meaning downregulation of expression of the respective gene).

Supplementary Figure 12.

Genes of the IPA canonical pathway "phase I – functionalization of compounds", "medium" concentration category

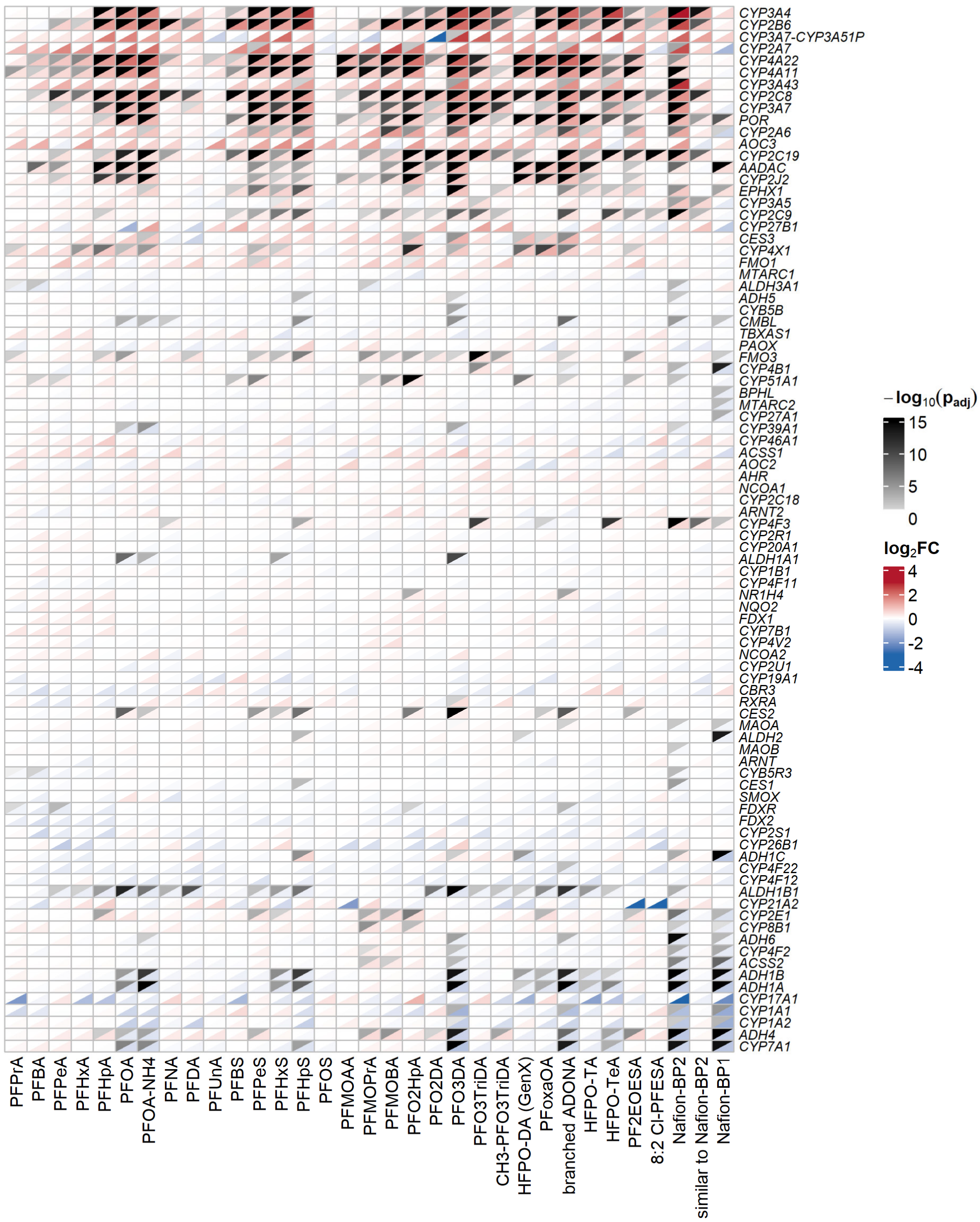

Supplementary Figure 12. Comparison analysis of the genes of the IPA canonical pathway "phase I – functionalization of compounds" based on the data from the "medium" concentration of the 33 PFAS. All genes that belong to the IPA-pathway "phase I – functionalization of compounds" are displayed. The genes are presented in the same order as for the "high" concentration category (Supplementary Figure 11). When $p_{adj} < 0.05$, it is presented in light-gray to black with increasing -$\log_{10}(p_{adj})$-values at the upper left triangles of the heatmap tiles. At the lower right triangles of the heatmap tiles, $\log_2$FC are given in red (meaning upregulation of expression of the respective gene) and blue (meaning downregulation of expression of the respective gene).

Supplementary Figure 13.

Genes of the IPA canonical pathway “phase I – functionalization of compounds”, “low” concentration category

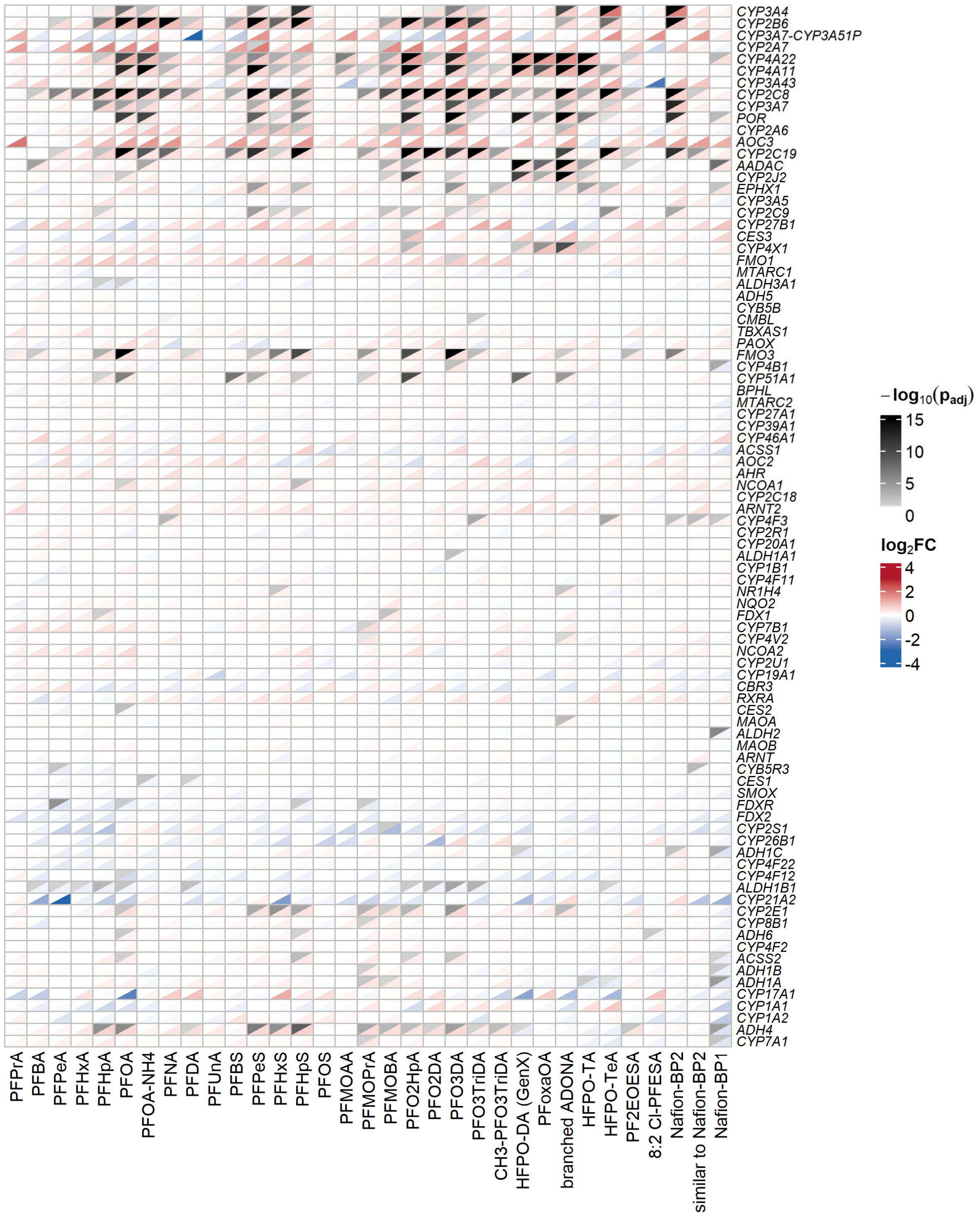

Supplementary Figure 13. Comparison analysis of the genes of the IPA canonical pathway “phase I – functionalization of compounds” based on the data from the “low” concentration of the 33 PFAS. All genes that belong to the IPA-pathway “phase I – functionalization of compounds” are displayed. The genes are presented in the same order as for the “high” concentration category (Supplementary Figure 11). When $p_{adj} < 0.05$, it is presented in light-gray to black with increasing -$log_{10}(p_{adj})$-values at the upper left triangles of the heatmap tiles. At the lower right triangles of the heatmap tiles, log_2FC are given in red (meaning upregulation of expression of the respective gene) and blue (meaning downregulation of expression of the respective gene).

Supplementary Figure 14.

Genes of the IPA canonical pathway “cholesterol biosynthesis”, “medium” concentration category

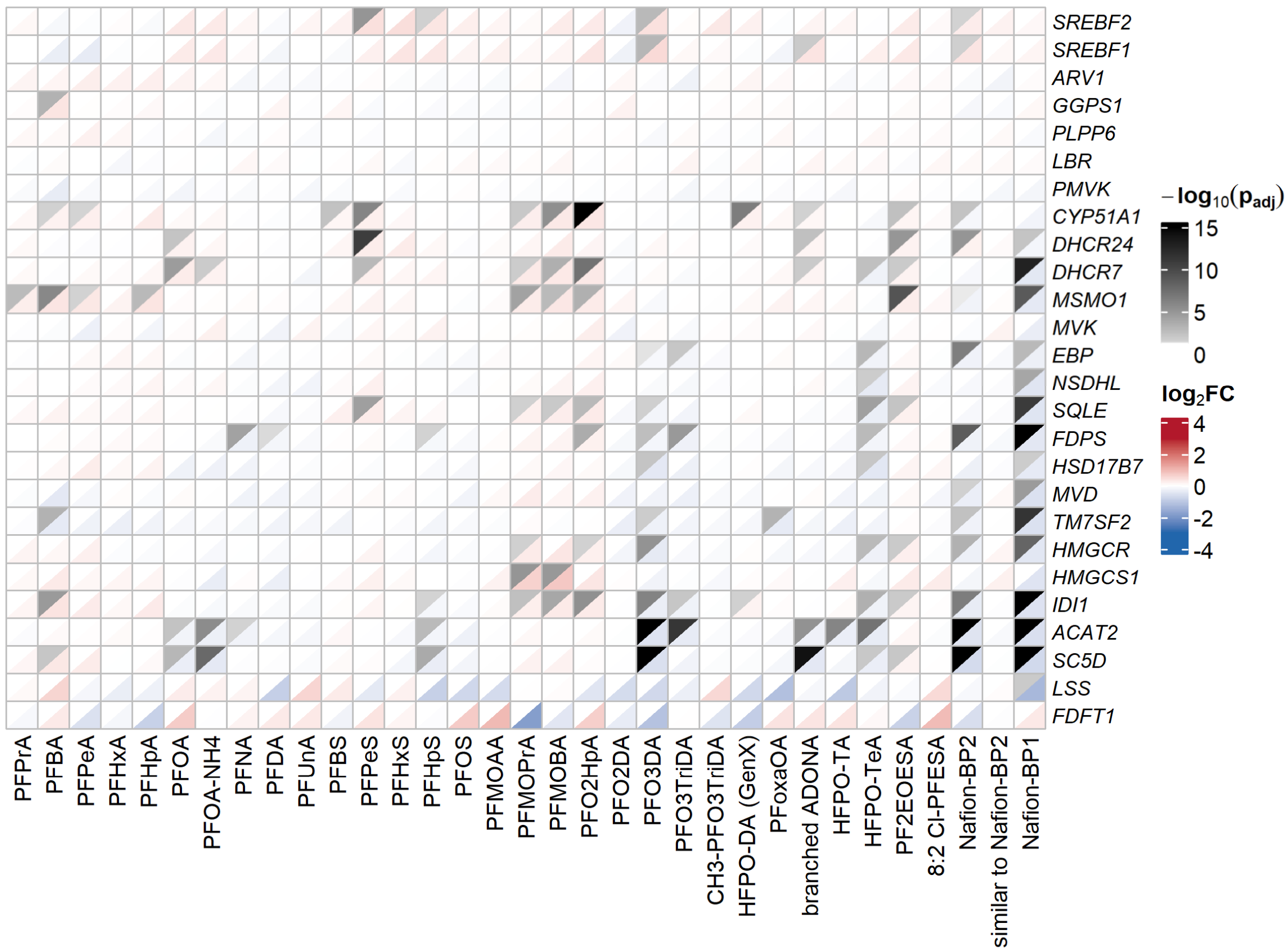

Supplementary Figure 14. Comparison analysis of the genes of the IPA canonical pathway “cholesterol biosynthesis” based on the data from the “medium” concentration of the 33 PFAS. All genes that belong to the IPA-pathway “cholesterol biosynthesis” are displayed. The genes were presented in the same order as for the “high” concentration category (Fig. 5). When $p_{adj} < 0.05$, it is presented in light-gray to black with increasing $-\log_{10}(p_{adj})$-values at the upper left triangles of the heatmap tiles. At the lower right triangles of the heatmap tiles, $\log_2FC$ are given in red (meaning upregulation of expression of the respective gene) and blue (meaning downregulation of expression of the respective gene).

Supplementary Figure 15.

Genes of the IPA canonical pathway “cholesterol biosynthesis”, “low” concentration category

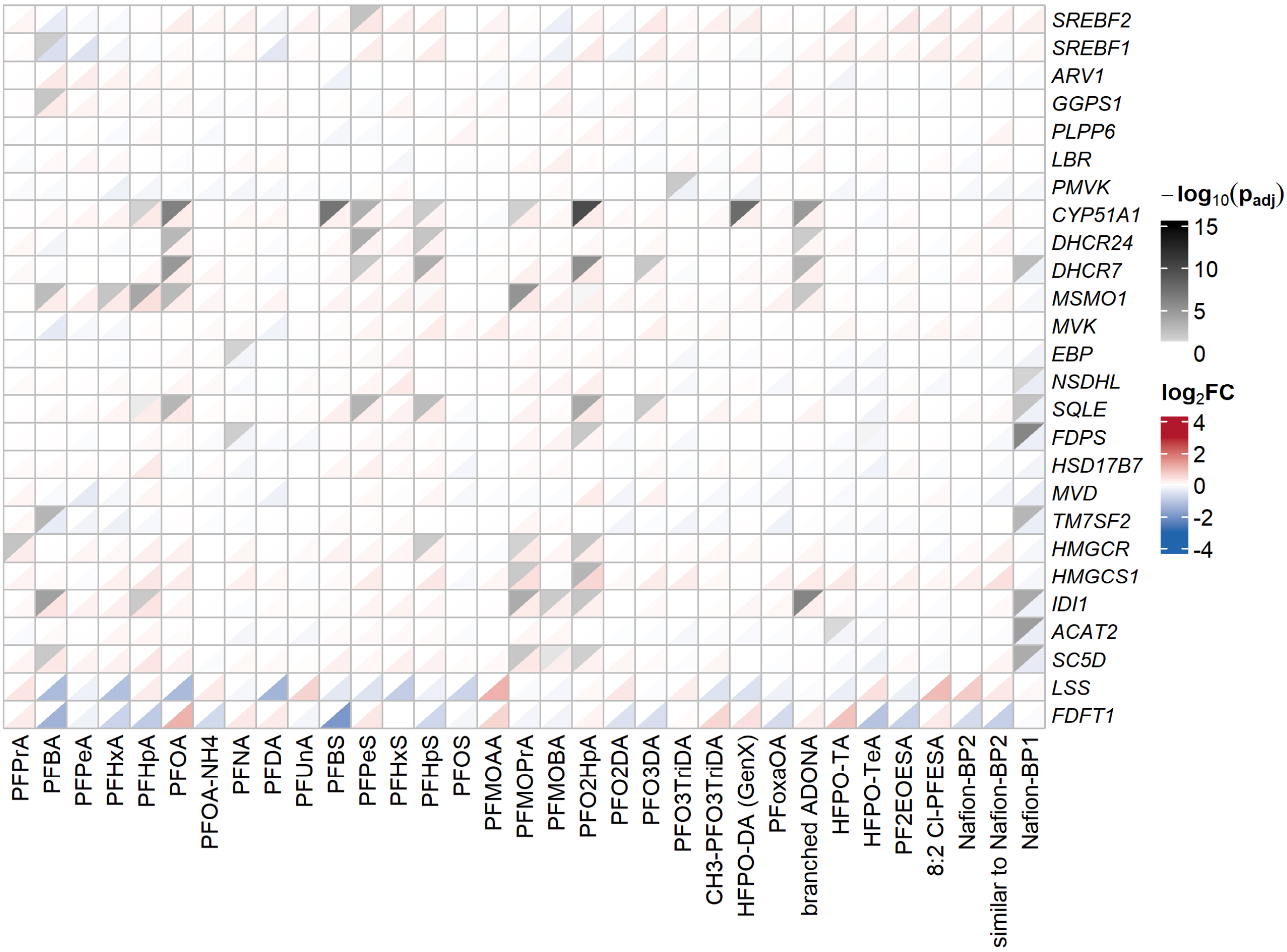

Supplementary Figure 15. Comparison analysis of the genes of the IPA canonical pathway “cholesterol biosynthesis” based on the data from the “low” concentration of the 33 PFAS. All genes that belong to the IPA-pathway “cholesterol biosynthesis” are displayed. The genes were presented in the same order as for the “high” concentration category (Fig. 5). When $p_{adj} < 0.05$, it is presented in light-gray to black with increasing $-\log_{10}(p_{adj})$-values at the upper left triangles of the heatmap tiles. At the lower right triangles of the heatmap tiles, $\log_2FC$ are given in red (meaning upregulation of expression of the respective gene) and blue (meaning downregulation of expression of the respective gene).

Supplementary Figure 16.

Genes of the IPA canonical pathway "bile acid and bile salt metabolism", "medium" concentration category

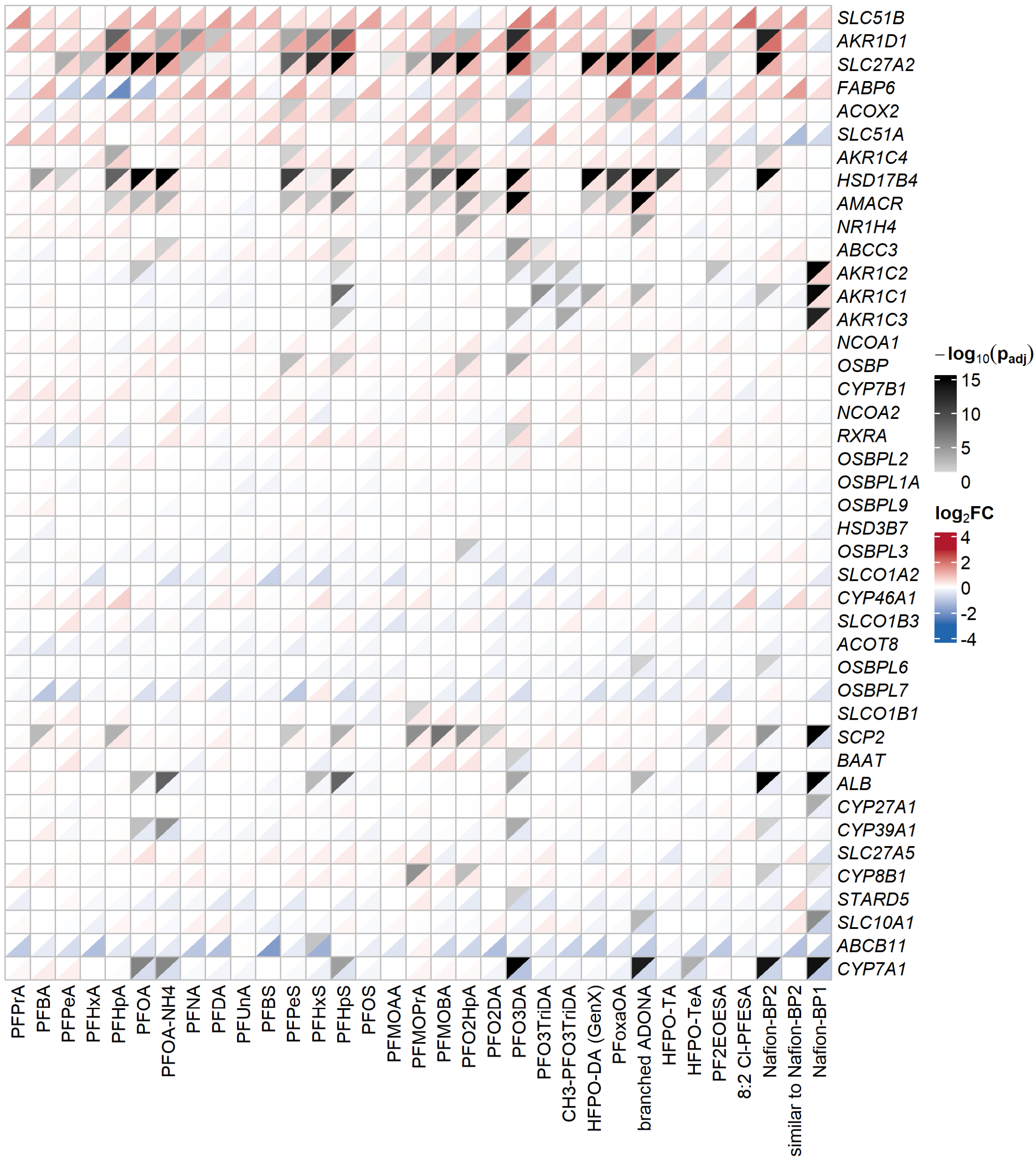

Supplementary Figure 16. Comparison analysis of the genes of the IPA canonical pathway "bile acid and bile salt metabolism" based on the data from the "medium" concentration of the 33 PFAS. All genes that belong to the IPA-pathway "bile acid and bile salt metabolism" are displayed. The genes were presented in the same order as for the "high" concentration category (Fig. 6). When $p_{adj} < 0.05$, it is presented in light-gray to black with increasing -$\log_{10}(p_{adj})$-values at the upper left triangles of the heatmap tiles. At the lower right triangles of the heatmap tiles, $\log_2$FC are given in red (meaning upregulation of expression of the respective gene) and blue (meaning downregulation of expression of the respective gene).

Supplementary Figure 17.

Genes of the IPA canonical pathway “bile acid and bile salt metabolism”, “low” concentration category

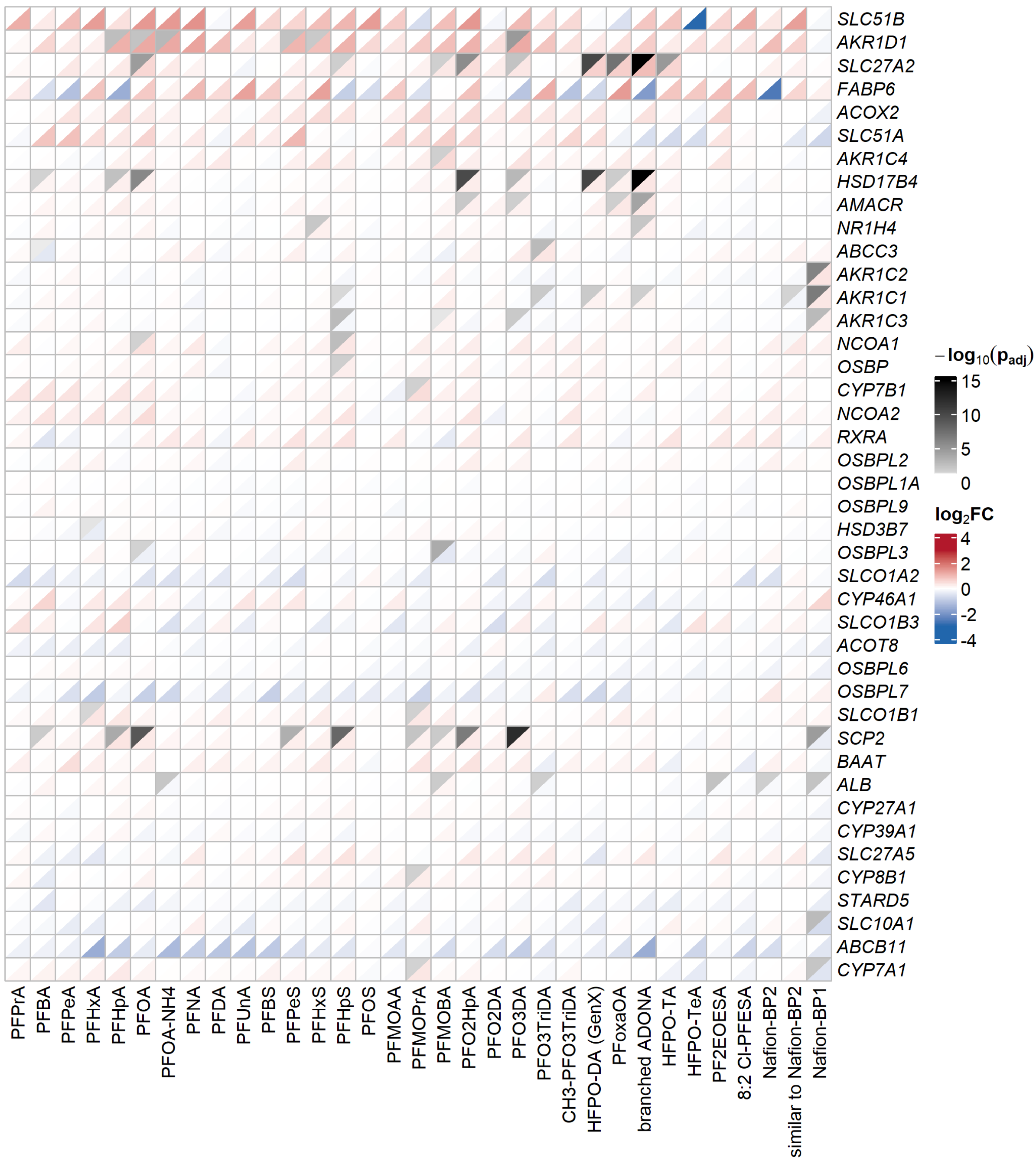

Supplementary Figure 17. Comparison analysis of the genes of the IPA canonical pathway “bile acid and bile salt metabolism” based on the data from the “low” concentration of the 33 PFAS. All genes that belong to the IPA-pathway “bile acid and bile salt metabolism” are displayed. The genes were presented in the same order as for the “high” concentration category (Fig. 6). When $p_{adj} < 0.05$, it is presented in light-gray to black with increasing $-\log_{10}(p_{adj})$-values at the upper left triangles of the heatmap tiles. At the lower right triangles of the heatmap tiles, $\log_2FC$ are given in red (meaning upregulation of expression of the respective gene) and blue (meaning downregulation of expression of the respective gene).
