# Supplementary material for: Analysis of transcriptomic alterations induced by 33 different per- and polyfluoroalkyl substances (PFAS) in differentiated HepaRG cells
Source: Arch Toxicol. 2026 May 13;100(9):4021–39. doi: 10.1007/s00204-026-04429-5 (PMC13454013; doi:10.1007/s00204-026-04429-5)
Supplement: Supplementary file 2 [file 204_2026_4429_MOESM2_ESM.pdf]

Analysis of transcriptomic alterations induced by 33 different per- and polyfluoroalkyl substances (PFAS) in differentiated HepaRG cells

Heike Sprenger[1], Wiebke Alker[1], Anna Rocchi[2], Chiara Leo[2], Rosa Giglio[2], Greta Immobile Molaro[2], Francesco Dondero[3], Albert Braeuning[1], Thorsten Buhrke[1#]

[1] German Federal Institute for Risk Assessment (BfR), Department Chemical and Product Safety, Max-Dohrn-Str. 8-10, 10589 Berlin, Germany

[2] NGS and Bioinformatic Laboratory – Polo d'Innovazione di Genomica, Genetica e Biologia SRL, Strada del Petriccio e Belriguardo 35-53100, Siena, Italy.

[3] Department of Science and Technological Innovation, University of Eastern Piedmont Alessandria Novara Vercelli, Viale Michel 11, 15121 Alessandria, Italy.

[#] Corresponding Author:

Dr. Thorsten Buhrke, German Federal Institute for Risk Assessment (BfR), Department Chemical and Product Safety, Max-Dohrn-Str. 8-10, 10589 Berlin, Germany; E-Mail thorsten.buhrke@bfr.bund.de, phone 0049 30 18412-77401

Supplementary Table 1

Supplementary Table 1. Names and structures of the PFAS used in the present study.

Group	Name	Abbreviation	Structure	CAS no.
PFCA	Perfluoropropionic acid	PFPrA	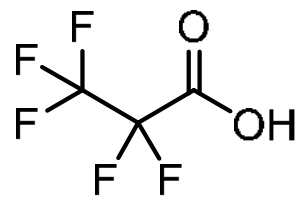	422-64-0
PFCA	Perfluorobutanoic acid	PFBA	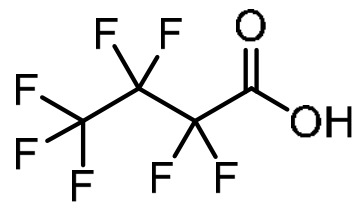	375-22-4
PFCA	Perfluoropentanoic acid	PFPeA	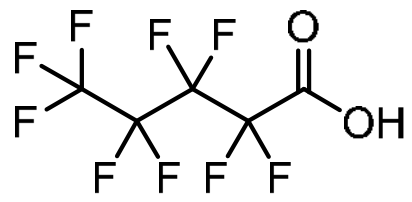	2706-90-3
PFCA	Perfluorohexanoic acid	PFHxA	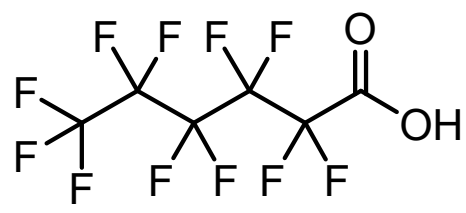	307-24-4
PFCA	Perfluoroheptanoic acid	PFHpA	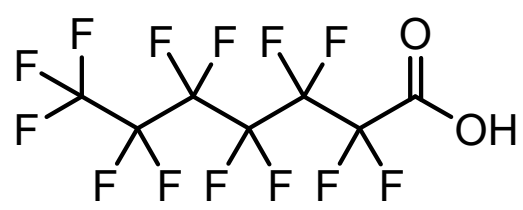	375-85-9
PFCA	Perfluorooctanoic acid	PFOA	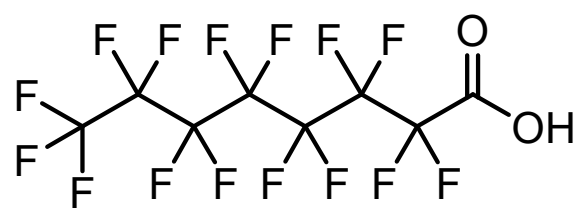	335-67-1
PFCA	Ammonium perfluorooctanoate	PFOA-NH4	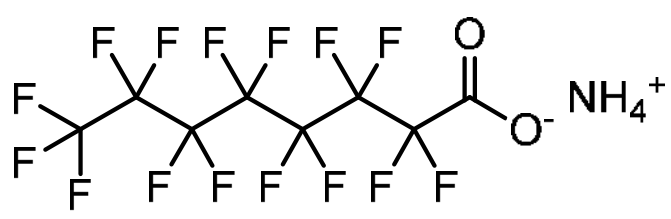	3825-26-1
PFCA	Perfluorononanoic acid	PFNA	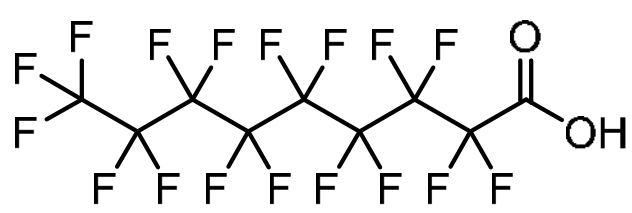	375-95-1
PFCA	Perfluorodecanoic acid	PFDA	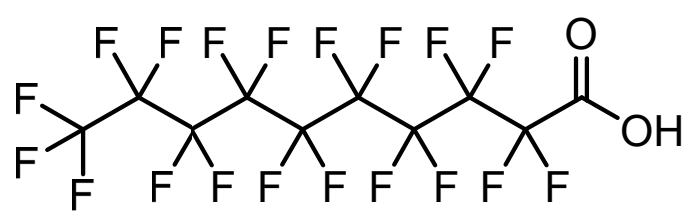	335-76-2
PFCA	Perfluoroundecanoic acid	PFUnA	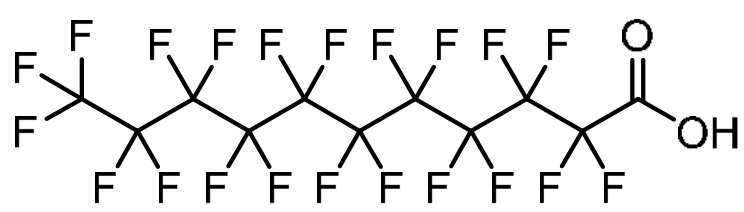	2058-94-8
PFSA	Perfluorobutanesulfonic acid	PFBS	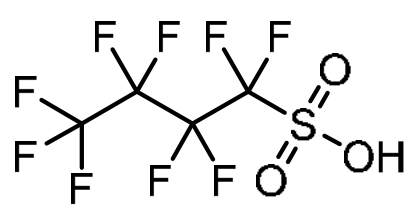	375-73-5
PFSA	Perfluoropentanesulfonic acid	PFPeS	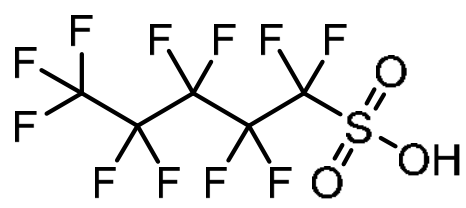	2706-91-4

PFSA	Perfluorohexanesulfonic acid potassium salt	PFHxS	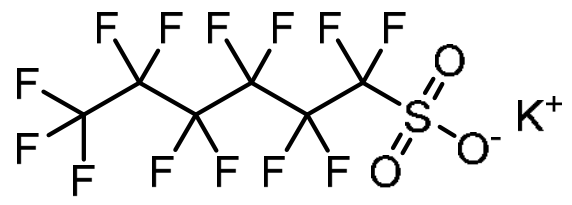	3871-99-6
PFSA	Perfluoroheptanesulfonic acid	PFHpS	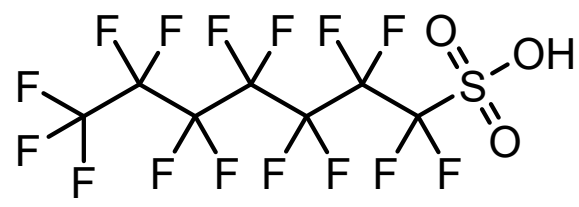	375-92-8
PFSA	Perfluorooctanesulfonic acid potassium salt	PFOS	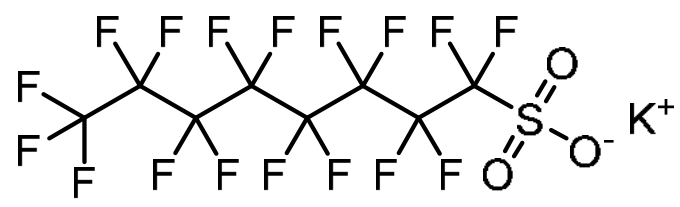	2795-39-3
PFECA linear monoether	2,2-difluoro-2-(trifluoromethoxy) acetic acid	PFMOAA	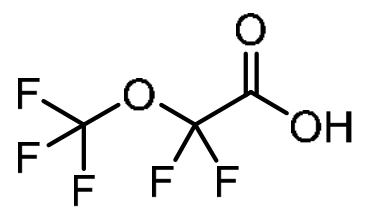	674-13-5
PFECA linear monoether	Perfluoro-3-methoxypropanoic acid	PFMOPrA	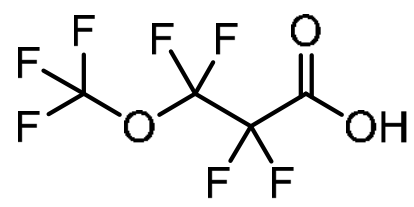	377-73-1
PFECA linear monoether	Perfluoro-4-methoxybutanoic acid	PFMOBA	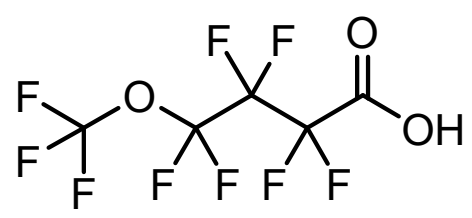	863090-89-5
PFECA linear polyether	Perfluoro-3,6-dioxaheptanoic acid	PFO2HpA	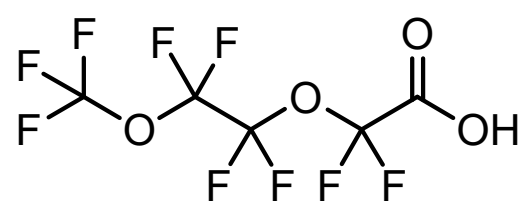	151772-58-6
PFECA linear polyether	Perfluoro-3,6-dioxadecanoic acid	PFO2DA	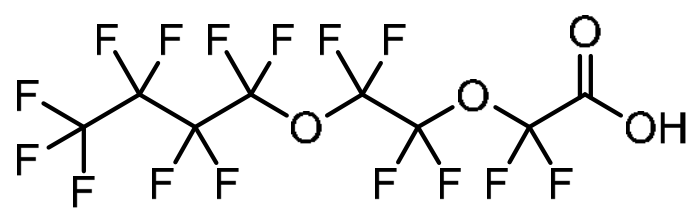	137780-69-9
PFECA linear polyether	Perfluoro-3,6,9-trioxadecanoic acid	PFO3DA	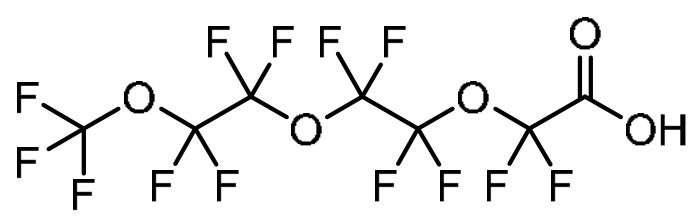	151772-59-7
PFECA linear polyether	Perfluoro-3,6,9-trioxatridecanoic acid	PFO3TriDA	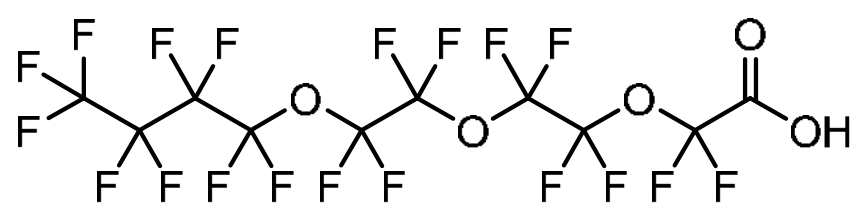	330562-41-9
PFECA linear polyether	Methyl perfluoro-3,6,9-trioxatridecanoate	CH3-PFO3TriDA	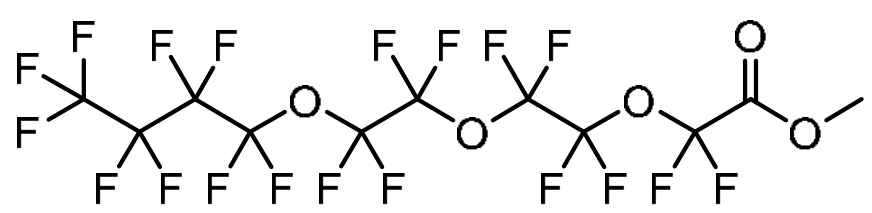	330562-42-0
PFECA branched monoether	2,3,3,3-Tetrafluoro-2-(heptafluoropropoxy)-propanoic acid	HFPO-DA (GenX)	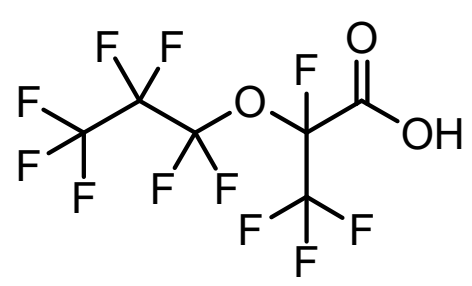	13252-13-6
PFECA branched monoether	Ammonium 2-perfluoropentoxy-2,3,3,3-tetrafluoropropanoate	PFoxaOA	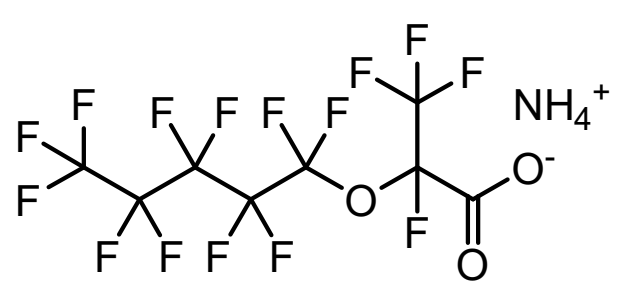	510774-81-9

PFECA branched polyether	Perfluoro-2,5-dimethyl-3,6-dioxaheptanoic acid	Branched ADONA	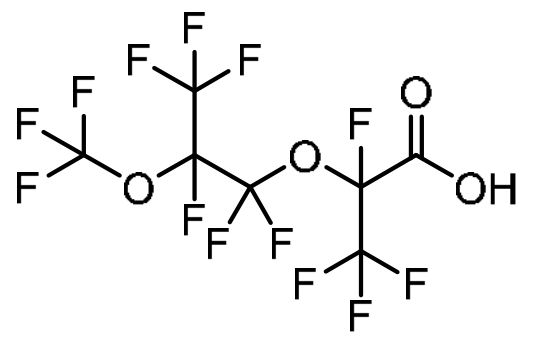	2479-73-4
PFECA branched polyether	Perfluoro-2,5-dimethyl-3,6-dioxaheptanoic acid	HFPO-TA	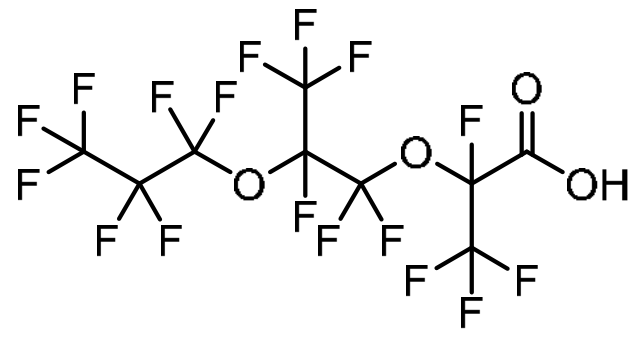	13252-14-7
PFECA branched polyether	Perfluoro-2,5,8-trimethyl-3,6,9-trioxadodecanoic acid	HFPO-TeA	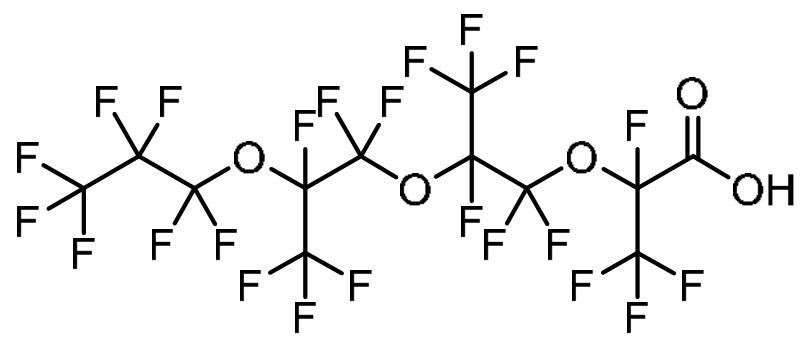	65294-16-8
PFESA linear monoether	Perfluoro-3-oxapentane-1-sulfonic acid	PF2EOESA	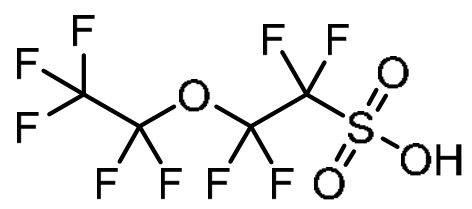	113507-82-7
PFESA linear monoether	11-chloroeicosafluoro-3-oxaundecane-1-sulfonic acid	8:2 Cl-PFESA	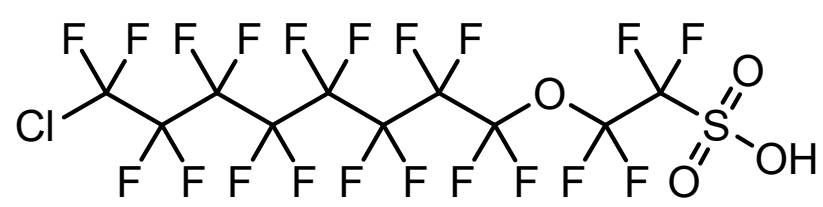	763051-92-9
PFESA branched polyether	7H-Perfluoro-4-methyl-3,6-dioxaoctanesulfonic acid	Nafion-BP2	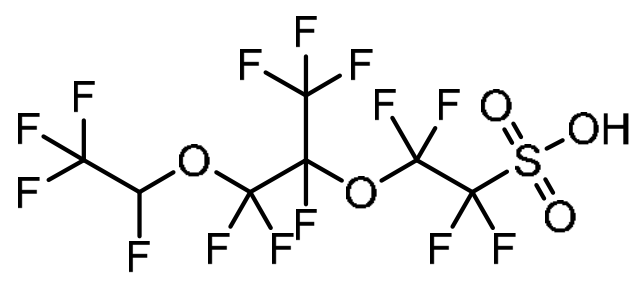	749836-20-2
PFESA branched polyether	Potassium perfluoro(4-methyl-3,6-dioxaoctane)-sulfonate	similar to Nafion-BP2	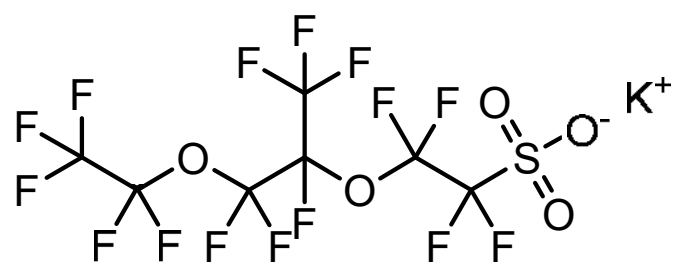	70755-50-9
PFESA branched polyether	Perfluoro-3,6-dioxa-4-methyl-7-octene-1-sulfonic acid	Nafion-BP1	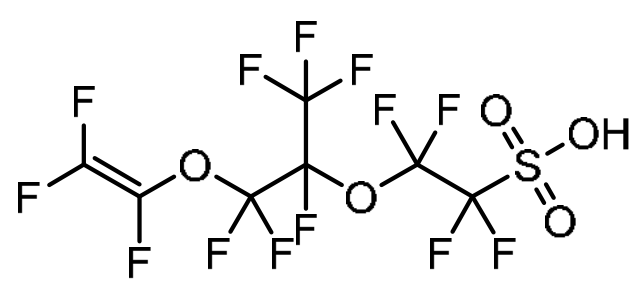	29311-67-9
